# Midday Dipping and Circadian Blood Pressure Patterns in Acute Ischemic Stroke

**DOI:** 10.3390/jcm12144816

**Published:** 2023-07-21

**Authors:** Nikolaos Kakaletsis, George Ntaios, Haralampos Milionis, Anastasia Karagiannaki, Ioanna Chouvarda, Vasiliki Dourliou, Ioannis Ladakis, Georgia Kaiafa, Konstantinos Vemmos, Christos Savopoulos

**Affiliations:** 1 First Propedeutic Department of Internal Medicine, Medical School, Aristotle University of Thessaloniki, AHEPA University Hospital, 54636 Thessaloniki, Greece; vicky_dourliou@hotmail.com (V.D.); gdkaiafa@yahoo.gr (G.K.); chrisavopoulos@gmail.com (C.S.); 2 Department of Internal Medicine, Faculty of Medicine, School of Health Sciences, University of Thessaly, 35100 Larissa, Greece; gntaios@med.uth.gr (G.N.); anastasiakaragiannaki@hotmail.com (A.K.); 3 Department of Internal Medicine, Medical School, University of Ioannina, University Hospital of Ioannina, 45500 Ioannina, Greece; hmilioni@uoi.gr; 4 Laboratory of Computing, Medical Informatics and Biomedical—Imaging Technologies, Aristotle University of Thessaloniki, 54124 Thessaloniki, Greece; ioannach@auth.gr (I.C.); ldk.gia@hotmail.com (I.L.); 5 Hellenic Stroke Organization, 11528 Athens, Greece; vemmosk@gmail.com

**Keywords:** acute ischemic stroke, midday dipping, nocturnal dipping, circadian blood pressure patterns, outcome

## Abstract

The purpose of this study was to investigate the alterations in blood pressure (BP) during midday and the changes in circadian BP patterns in the acute phase of ischemic stroke (AIS) with the severity of stroke and their predictive role outcomes within 3 months. A total of 228 AIS patients (a prospective multicenter follow-up study) underwent 24 h ambulatory blood pressure monitoring (ABPM). Mean BP parameters during the day (7:00–22:59), the midday (13:00–16:59), and the night (23:00–6:59), and midday and nocturnal dipping were calculated. Midday SBP dippers had less severe stroke, lower incidence of hypertension and SBP/DBP on admission, lower levels of serum glucose and WBCs, and delayed initiation of ABPM compared to risers. There was a reverse relation between midday SBP dipping and both nocturnal dipping and stroke severity. The “double dippers” (midday and nocturnal dipping) had the least severe stroke, the lowest SBP/DBP on admission, the lowest heart rate from ABPM, and a lower risk of an unfavorable outcome, while the “double risers” had the opposite results, by an approximately five-fold risk of death/disability at 3 months. These findings indicate different circadian BP patterns during the acute phase of AIS, which could be considered a marker of stroke severity and prognosis.

## 1. Introduction

A siesta (a short afternoon nap) is a common practice, probably due to climatic or social factors, in Mediterranean and Latin American countries and in China [1,2], and it seems to become more common with increasing age [3]. Initial studies from Greece suggested that a siesta may have a protective effect on cardiovascular events [4,5,6], while other reports from Israel and Costa Rica indicated that it increases the incidence and mortality of coronary artery disease [7,8,9]. Effects of a siesta on blood pressure (BP) are well known [10], as previous studies indicated a decline in blood pressure during the siesta comparable with that occurring during night sleep, and thus, it may compose a remarkable part of the BP circadian rhythm [11,12].

Generally, circadian rhythms are daily cycles of physiology and behavior with a period of approximately (circa) one day (diem) [13,14] and play important roles in the homeostasis of the human body [15]. It is known that blood pressure presents a circadian pattern that is characterized by higher diurnal values followed by a nocturnal decrease in both systolic and diastolic BP while sleeping (dipping) [16]. This typical circadian BP pattern, in addition to this profound sleep-related BP decline, also exhibits two daytime peaks, the first around or just after the commencement of daytime activity (morning surge) and a second, typically of smaller magnitude, a few hours later, and in between, there is a small afternoon nadir (approximately the duration of a siesta) [17]. These variations are determined not only by the sleep–activity cycle, but also by several endogenous circadian rhythms that affect vascular and cardiac functions [18].

Any disruption in circadian activity may predispose individuals to the disequilibrium of the cardiovascular system. These disruptions are the factors associated with abnormal daytime and night-time BP and nocturnal dipping [16] and maybe BP during midday. Thus, in some individuals, during sleep, the nocturnal dipping is blunted or reversed. This results in the absence of a nocturnal dip (“dipper”) or even a slight increase in blood pressure above the wake-time level, termed “non-dipper” and “reverse-dipper or riser” [19,20]. Studies have found that these abnormal blood pressure patterns are linked to an increased risk for cardiovascular events and target organ damage in both normotensive and hypertensive individuals [21,22,23,24]. Moreover, better control of sleep blood pressure has been shown to exert greater protection against stroke and cardiovascular events in many studies [25,26]. However, there are scarce data concerning the predictive role of circadian blood pressure patterns after acute ischemic stroke (AIS) and no data about the alteration of BP during midday and its predictive role compared to nocturnal values.

The purpose of this study is to investigate the variation in BP levels during midday and the changes in circadian blood pressure patterns after acute ischemic stroke through 24 h ambulatory blood pressure monitoring (ABPM) methods, during the acute phase of AIS, with the severity of stroke and their predictive value for medium-term (3-month) outcomes.

## 2. Materials and Methods

### 2.1. Study Design, Study Population, and Selection Criteria

For the purpose of this study, we used data obtained from the PREVISE study (NCT01915862) [27], which investigated the predictive role of BPV indices derived from 24 h ABPM methods in AIS patients, and its methodology was previously described in detail [28]. Briefly, this was a prospective follow-up study conducted in three university hospitals in northern, central, and western Greece. All study patients were prospectively admitted to hospitals with acute ischemic stroke (<24 h after stroke onset) and evaluated on admission. Medical history, clinical profile, cardiovascular risk factors, acute management modalities, and laboratory findings on admission were recorded. The severity of stroke on admission was assessed according to the National Institute of Health’s stroke scale (NIHSS) [29]. The study was approved by the local ethics and scientific committee, and all patients or their relatives signed an informed consent.

### 2.2. Twenty-Four-Hour BP Monitoring

The 24 h ABPM was performed with the automated oscillometric device TM2430 (A&D Company Ltd., Tokyo, Japan), which is validated and recommended by the European Hypertension Society [30,31]. Implementation of the oscillometric device and all procedures were carried out according to the European Society of Hypertension Guidelines on Ambulatory Blood Pressure Monitoring [20]. The nonparetic arm was chosen for all BP recordings, or the right arm in case of coma or tetraparesis. Blood pressure measurements were obtained with 24 h ABPM every 20 min within 48 h of stroke onset during the daytime (7:00–22:59) and night-time (23:00–6:59). An acceptable 24 h ABPM recording for our study should have had ≥50 (70%) acceptable measurements (and at least 20 and 7 acceptable measurements during day- and night-time, respectively).

There was not any predefined protocol concerning the antihypertensive therapy during the acute phase of stroke. The management of BP during the acute phase of ischemic stroke was performed according to the recent European guidelines, which recommend against routine BP lowering in acute stroke and suggest that cautious BP lowering should be initiated in patients with extremely high BP > 220/120 mmHg or 185/110 mmHg in the case of thrombolysis [32]. Thus, the antihypertensive treatment and the decision to discontinue or continue the previous antihypertensives (AHTs) was at the discretion of the attending physician according to these guidelines and the local hospital pathways.

### 2.3. Outcome Measures

The modified Rankin Scale (mRS) was used to assess neurological function at 3 months after admission [33] with an onsite follow-up re-examination or with telephone follow-up by a physician certified with mRS evaluation. Endpoints were death (mRS = 6) at 3 months in all stroke patients who met the inclusion criteria and unfavorable functional outcome (disability/death) (mRS > 2) at 3 months in the subgroup of patients with no prior disability (pre-stroke mRS < 3).

### 2.4. Definition of Nocturnal and Midday Dipping, Dipping Status, and Blood Pressure Circadian Patterns Derived from ABPM

The mean systolic (SBP) and diastolic BP (DBP) were calculated for 24 h, for daytime (7:00–22:59), night-time (23:00–6:59), midday time (13:00–16:59), and day-without-midday-time (7:00–12:59 and 17:00–22:59). We also calculated the mean heart rate (HR) for 24 h, daytime and night-time.

Nocturnal dipping was calculated by the following formula:

$$Nocturnal\;dipping=\frac{{BP}_{day\_time}-{BP}_{night\_time}}{{BP}_{day\_time}}\times 100.$$

Midday dipping was calculated by the following formula:

$$Midday\;dipping=\frac{{BP}_{day\_without\_midday\_time}-{BP}_{midday\_time}}{{BP}_{day\_without\_midday\_time}}\times 100.$$

Nocturnal and midday dipping (units: %) were calculated for SBP and DBP. We divided patients into dippers and non-dippers (nocturnal, midday, SBP, DBP) if they had dipping >0% and ≤0%, respectively.

We distinguished four different BP circadian patterns according to patients’ midday and nocturnal systolic BP dipping status: 1. Midday and nocturnal dipping; patients’ dippers during midday (midday SBP dipping > 0%) and also dippers during night (nocturnal SBP dipping > 0%). 2. Midday dipping and nocturnal rise; patients’ dippers during midday (midday SBP dipping > 0%) and non-dippers during night (nocturnal SBP dipping ≤0%). 3. Midday rise and nocturnal dipping; patients’ non-dippers during midday (midday SBP dipping ≤ 0%) and dippers during night (nocturnal SBP dipping > 0%). 4. Midday and nocturnal rise; patients’ non-dippers during midday (midday SBP dipping ≤ 0%) and also non-dippers during night (nocturnal SBP dipping ≤0%). As ABPM was for 24 h (starting time varied—within 48 h of stroke onset), in each patient, it corresponded to one midday and one night period and could be included in only one BP circadian pattern group. Patients’ baseline data, BP, and HR parameters derived from ABPM, and outcomes were compared across the four groups of BP circadian patterns.

Additionally, mean values and the dipping of blood pressure of SBP, DBP, and HR parameters derived from ABPM were compared across the three groups of stroke severity (minor stroke, NIHSS: 0–7; moderate stroke, NIHSS: 8–16; severe stroke, NIHSS: >16).

### 2.5. Statistical Analysis

The non-parametric Mann-Whitney U test was used to compare differences between mean values of two continuous variables and the non-parametric Kruskal–Wallis test was used to compare differences between mean values of more than two continuous variables. Comparisons of categorical variables were performed with Pearson’s Chi-Square test or Fisher’s Exact test. Spearman’s rank correlation analysis was performed to analyze the correlation between midday and nocturnal BP dipping and the correlation of stroke severity (NIHSS) with mean BP, HR, and BP dipping, and Spearman’s rank correlation coefficient was calculated. In order to evaluate the predictive role of midday dipping and each one of the four SBP circadian patterns, we performed univariate logistic regression analysis.

All other statistical tests were 2-sided, and a *p*-value less than 0.05 was taken as the level of statistical significance. The editing statistics were performed with IBM SPSS Statistics for Windows (Version 25.0. Armonk, NY, USA: IBM Corp. Released 2017). Downloading and editing of BP data from ABPM device were performed with Doctor Pro 3 (A&D Company Ltd., Tokyo, Japan).

## 3. Results

Out of 228 patients who participated in this study, 175 had no prior disability (pre-stroke mRS < 3). Their mean age was 80 (±7.1) years, 45.6% were male, and 83.8% had a history of arterial hypertension. Baseline clinical and laboratory findings of study participants and their BP and HR parameters derived from 24 h ABPM in total and separately according to midday and nocturnal dipping status of SBP and DBP are depicted in detail in Supplementary S1 and S2.

Compared to non-dippers, midday SBP dippers had less severe strokes, lower incidence of hypertension and SBP/DBP on admission, and lower levels of serum glucose and WBC, but longer duration between stroke onset and the initiation of ABPM. On the contrary, nocturnal SBP dippers did not differ in their baseline clinical and laboratory characteristics compared to nocturnal SBP non-dippers (Table 1, Supplementary S1; Table S1).

Midday DBP dippers had a higher incidence of heart failure and previous stroke, as well as administration of BP-lowering agents during the acute phase of stroke and longer duration between stroke onset and the initiation of ABPM and also lower levels of serum glucose and SBP on admission and less severe stroke, compared to midday DBP non-dippers. Similarly, nocturnal DBP dippers also had less severe stroke compared to nocturnal DBP non-dippers and lower DBP on admission and platelet count (Supplementary S1; Table S3). Similar results were found in the unfavorable outcome analysis of the 175 patients without prior disability (Supplementary S2; Tables S10 and S12).

**Figure 1:**
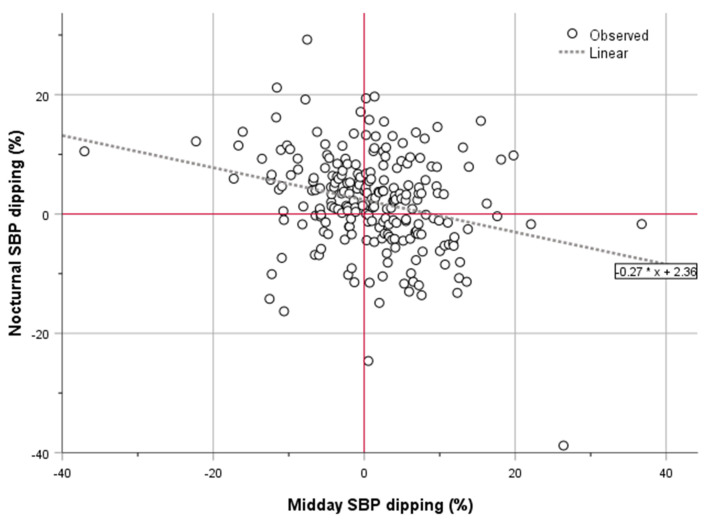
Scatterplot of the relationship between midday and nocturnal SBP dipping derived from the ABPM of all study participants.

Mean heart rate values were always lower during 24 h ABPM, daytime, and night-time in SBP midday and nocturnal dippers. Midday SBP/DBP dippers had lower BP nocturnal dipping or a reverse in BP nocturnal dipping, and nocturnal SBP/DBP dippers had lower BP midday dipping or a reverse in BP midday dipping (Supplementary S1; Tables S2 and S4,  Supplementary S2; Tables S11 and S13). This reverse relation of midday with nocturnal dipping was obvious but weak in correlation analysis and in the relative scatterplots (Figure 1, Supplementary S1; Figure S1) for both SBP and DBP (Supplementary S1; Table S5) in all stroke patients, and only for SBP in patients without prior disability (Supplementary S2; Table S14 and Figure S3).

In the death outcome analysis, out of 228 patients, 61 died during the 3-month follow-up (incidence rate: 26.7, 95% CI: 20.9–32.5). Only nocturnal DBP dippers had a lower mortality rate (Supplementary S1; Table S3). In the unfavorable outcome analysis, of the 175 patients without prior disability, 79 finally met the endpoint of disability/death during the 3-month follow-up (incidence rate: 45.1, 95% CI: 37.7–52.5). Both midday and nocturnal SBP dippers (without prior disability) had an approximately 20% lower mortality rate, and only nocturnal DBP dippers had a lower mortality rate compared to non-dippers (Supplementary S2; Tables S10 and S12).

By comparing mean values and dipping of BP and HR from ABPM across the three groups of stroke severity (minor stroke, NIHSS: 0–7; moderate stroke, NIHSS: 8–16; severe stroke, NIHSS: >16), we found that only midday SBP dipping and HR were linearly associated with the severity of stroke. This means that more severe strokes were associated with a lower BP midday dipping or a reverse (rise) in BP midday dipping and higher heart rate (Supplementary S1; Tables S6 and S7). Similar and more robust results were found in the unfavorable outcome analysis of the 175 patients without prior disability (Supplementary S2; Tables S15 and S16).

**Figure 2:**
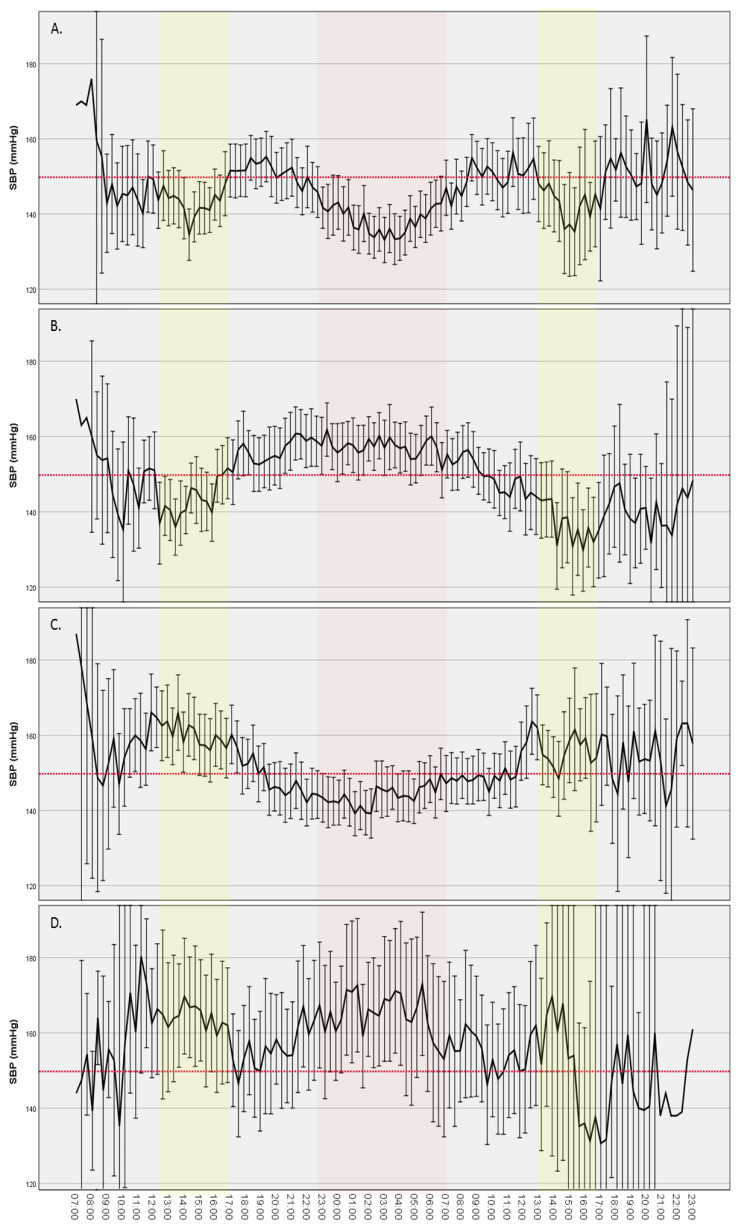
Circadian variation in mean systolic blood pressure (SBP) with 95% CI of all study participants in the four different SBP circadian pattern groups: (**A**) midday and nocturnal dipping (“double dippers”); (**B**) midday dipping and nocturnal rise; (**C**) midday rise and nocturnal dipping, and (**D**) midday and nocturnal rise (“double risers”). Yellow: Midday time (13:00–16:59); Red: Night-time (23:00–6:59); Red dashed line: Mean systolic blood pressure of all study participants without prior disability (149.8 mmHg).

By comparing baseline clinical and laboratory findings of study participants and their BP and HR parameters derived from 24 h ABPM among the four different systolic BP circadian patterns (Figure 2 and Supplementary S1; Figure S2), we found that the severity of stroke (NIHSS), SBP, and DBP on admission and HR from ABPM rose successively in patients with double midday/nocturnal dipping compared to patients with reverse midday/night dipping compared to double midday/nocturnal rising.

Patients with both midday and nocturnal dipping had the least severe stroke, the lowest SBP and DBP on admission, and HR from ABPM, and patients with both midday and nocturnal rising had the most severe stroke, the highest SBP and DBP on admission, and HR from ABPM. All other patients had intermediate values of the above variables (Supplementary S1; Tables S8 and S9). Similar results were found in the unfavorable outcome analysis of the 175 patients without prior disability (Supplementary S2; Tables S17 and S18).

There was no statistically significant difference in mortality rate at 3 months after stroke onset among the four different BP circadian patterns (Table 2, Supplementary S1; Table S8).

However, in unfavorable outcome analysis (patients without prior disability), patients with both midday and nocturnal dipping had the lowest disability/death rate (29.3%), and patients with both midday and nocturnal rising had the highest disability/death rate (77.8%, *p* = 0.003). In all other BP circadian patterns, the unfavorable outcome rate was approximately 50% (Table 2, Supplementary S2; Table S17). Furthermore, univariate logistic regression analysis revealed that “double dippers” had a 64% lower risk of an unfavorable outcome at three months, while “double risers” had an approximately five-fold risk of death/disability at three months (Table 3). However, in multivariate logistic regression analysis, with all statistically significant variables from univariate analysis (age, atrial fibrillation, admission NIHSS, stroke onset—ABPM initiation time, and WBC), according to a previous study [28], revealed that the predictive values of the above BP circadian patterns were not finally independent.

## 4. Discussion

While patients with AIS who were nocturnal SBP dippers did not differ in their baseline clinical and laboratory characteristics from risers, midday dippers of SBP had a lower incidence of hypertension, less severe stroke, lower SBP/DBP on admission, lower levels of serum glucose and WBCs, and longer duration between stroke onset and the initiation of ABPM compared to midday SBP risers. Additionally, midday DBP dippers also had a higher incidence of heart failure, previous stroke, and the administration of BP-lowering agents during the acute phase of stroke. This indicates that during the acute phase of AIS, the expected disturbance of BP dipping is more obvious during midday than at night-time.

Except for the anticipated differences in patients’ clinical history (hypertension, heart failure, previous cerebrovascular disease), patients’ physical stress that initially originated from the severity of the disease plays a major role (stroke; NIHSS), assisted by the earlier initiation of ABPM, and was finally indicated with a higher level of serum glucose and WBC, which are well known to be associated with higher disease severity and physical stress. Stress and inflammatory response are the two main causative factors leading to higher WBC levels and high glucose in stroke patients through a highly complex interplay of counter-regulatory hormones such as catecholamines, growth hormone, cortisol, and cytokines [34,35]. The above hypothesis is enhanced by the observed respective differences in ABPM heart rate, which are an important marker of stress-induced vascular damage [36,37].

In contrast with previous studies, where BP fall during daytime sleep was strongly correlated with nocturnal BP fall (the dipping pattern was consistent during the day and night-time sleep in two-thirds of cases) [2], paradoxically, we found a weak but reverse relation of midday dipping with nocturnal dipping. This could be attributed to possibly disturbed BP circadian patterns after AIS or the absence of midday sleep (siesta), or it may be an expected finding due to the definition and the formula we used to calculate the nocturnal dipping, which also included midday BP values in the daytime measurements.

However, only midday SBP dipping (and HR) was found to be linearly associated with the severity of stroke. This means that more severe strokes were associated with lower BP midday dipping or a reverse (rise) in BP midday dipping.

In general, most patients with AIS are non-dippers, reverse dippers, and less extreme dippers, and normal dippers [38]. The loss of the expected nocturnal BP dipping in AIS has been reported in several studies [39,40,41,42,43]. Normal BP nightfall depends, in part, on changes in the activity of the sympathetic nervous system (SNS) [44]. The loss of dipping and the increase in nocturnal BP are associated with an increase in norepinephrine plasma levels, suggesting an alteration in SNS activity [43]. This elevated SNS activity during the night may determine the dipping status in non-dippers and reverse dipper patients [45,46]. The presence or the loss of midday dipping may be controlled by the same pathophysiological pathways.

In the death outcome analysis, only nocturnal DBP dippers had a lower mortality rate. In unfavorable outcome analysis, both midday and nocturnal SBP dippers (without prior disability) had approximately 20% lower mortality rate, and again, only nocturnal DBP dippers had a lower mortality rate than non-dippers.

In line with our results, the long-term risk of death was found to be greater in AIS patients who had a reversal of the normal characteristics of the 24 h BP with higher values at night than during the day, irrespective of stroke severity [47]. Another study of patients with first-ever AIS showed that an increase in BP dipping favors optimum short-term outcomes [48]. Moreover, in a study of patients with AIS confirmed using computerized brain tomography, the absence of a change in SBP and DBP during the night was independently associated with poor outcomes (mRS > 2) [49]. However, there is also a study in which the abnormal BP nightfall was not associated with adverse outcomes at 3 months [50]. Concerning the midday BP dipping, there are no data to date.

Finally, we found a sequential deterioration/disturbance between patients with double midday/nocturnal dipping compared to patients with reverse midday/night dipping compared to double midday/nocturnal rising, reflected by the severity of stroke, SBP and DBP on admission, and HR. The “double dippers” had the least severe stroke, the lowest SBP/DBP on admission, and HR from ABPM, while the “double risers” had the opposite. These findings also may indicate a sequential alteration in SNS activity according to stroke severity and patients’ physical stress and thus their circadian BP patterns.

Even though we did not find differences in mortality rate among the four different BP circadian patterns, in the unfavorable outcome analysis (in patients without prior disability), “double dippers” had the lowest disability/death rate, and “double risers” had the highest disability/death rate. Furthermore, these diametrically opposed BP circadian patterns were associated with favorable and unfavorable 3-month outcomes, respectively, but not independently.

We found only one previous study that investigated the changes in circadian blood pressure patterns after thromboembolic and hemodynamic brain infarction and evaluated the relation between circadian blood pressure variation, infarct location, and the activation of the autonomic nervous system. This study found that patients with thromboembolic hemispheric infarction had a pathologically reduced or abolished circadian blood pressure variation (without the biphasic circadian BP pattern) and a significantly higher norepinephrine concentration than the patients who maintained the nocturnal reduction in blood pressure [43].

The main limitation of this study is that we did not record the actual patients’ sleeping time, but we used an arbitrary night and midday (siesta) time when we hypothesized that patients were sleeping or at least resting in their beds. Thus, in no way could we attribute the observed finding to sleep; rather, we could attribute this finding to alterations in circadian rhythms due to endogenous or exogenous reasons in the acute phase of AIS. It would be of great interest for future studies to investigate whether these PB circadian patterns are associated with abnormal circadian variations in established circadian biomarkers (melatonin, cortisol) [51]; melatonin is generally not affected by the sleep–wake cycle or stress factors when cortisol is influenced by environmental factors and stroke characteristics [52]. Another limitation is that most patients in our study were not treated with either thrombolysis or mechanical cerebral reperfusion. This issue, of course, limits the generalizability of the results to the wider population of patients with AIS. The main strength of the present study is that it is a prospective multi-center observational study with a standard protocol that involves many consecutive BP measurements (every 20 min) per patient during the acute phase of stroke using the same type of ABPM device in all three sites.

## 5. Conclusions

This is the first study that investigates the alterations in blood pressure during midday in the acute phase of ischemic stroke and its association with nocturnal dipping, the severity of stroke, and the differences among the different BP circadian patterns according to midday and nocturnal dipping and their predictive role for the 3-month outcome. Our findings indicate the existence of different circadian BP patterns according to stroke severity in the acute phase of AIS that might be related to patients’ physical stress and alterations in their sympathetic nervous system activity, which should be considered during the acute management of blood pressure in AIS. The clinical value of these findings should be validated in future studies.

## Figures and Tables

**Table 1:** Baseline clinical and laboratory findings and characteristics of study participants according to SBP dipping status.

	Total (n = 228)	Midday SBP Dipping Status			Nocturnal SBP Dipping Status		
		Non-Dippers ≤ 0% (n = 95)	Dippers > 0% (n = 132)	*p*	Non-Dippers ≤ 0% (n = 86)	Dippers > 0% (n = 142)	*p*
Age (years)	80.0 ± 7.1	80.0 ± 7.0	80.0 ± 7.1	0.871	80.6 ± 6.9	79.6 ± 7.1	0.450
Sex (male)	104 (45.6%)	40 (42.1%)	64 (48.5%)	0.341	39 (45.3%)	65 (45.8%)	0.950
Pre-Stroke mRS > 2	51 (22.6%)	24 (25.3%)	26 (20.0%)	0.348	20 (23.8%)	31 (21.8%)	0.731
NIHSS admission	7 (13)	10 (14)	5 (12)	0.002 *	9 (12)	6 (12)	0.179
Hypertension	191 (83.8%)	85 (89.5%)	105 (79.5%)	0.046 *	69 (80.2%)	122 (85.9%)	0.259
Diabetes	71 (31.1%)	28 (29.5%)	43 (32.6%)	0.619	24 (27.9%)	47 (33.1%)	0.412
Dyslipidemia	93 (40.8%)	44 (46.3%)	49 (37.1%)	0.165	31 (36.0%)	62 (43.7%)	0.257
Atrial Fibrillation	89 (39.0%)	39 (41.1%)	50 (37.9%)	0.629	38 (44.2%)	51 (35.9%)	0.215
Coronary Artery Disease	45 (19.7%)	17 (17.9%)	28 (21.2%)	0.536	20 (23.3%)	25 (17.6%)	0.299
Heart Failure	20 (8.8%)	8 (8.4%)	12 (9.1%)	0.861	5 (5.8%)	15 (10.6%)	0.219
Previous Stroke	86 (37.7%)	32 (33.7%)	54 (40.9%)	0.268	32 (37.2%)	54 (38.0%)	0.902
SBP admission (mmHg)	154.6 ± 27.0	162.0 ± 29.3	149.8 ± 23.9	0.003 *	155.7 ± 26.7	154.0 ± 27.2	0.887
DBP admission (mmHg)	84.3 ± 16.2	87.3 ± 17.3	82.2 ± 15.2	0.034 *	86.1 ± 18.0	83.2 ± 15.1	0.363
HR admission (bpm)	77.7 ± 15.6	79.5 ± 16.7	76.4 ± 14.6	0.307	78.1 ± 15.5	77.4 ± 15.6	0.714
Onset-ABPM (hours)	18.8 ± 9.8	17.2 ± 9.8	20.0 ± 9.7	0.037 *	18.6 ± 10.0	19.0 ± 9.6	0.855
Glucose (mg/dL)	135.0 ± 47.8	140.9 ± 52.5	131.0 ± 43.9	0.046 *	134.8 ± 50.6	135.2 ± 46.3	0.757
eGFR (mL/min/1.73m^2^)	63.1 ± 19.4	61.3 ± 19.0	64.3 ± 19.6	0.290	65.2 ± 18.6	61.9 ± 19.8	0.204
WBC (K/μL)	8.6 ± 3.0	9.3 ± 3.4	8.2 ± 2.5	0.014 *	8.2 ± 2.1	8.9 ± 3.4	0.621
Hematocrit (%)	39.4 ± 4.6	39.3 ± 4.3	39.5 ± 4.9	0.663	39.7 ± 4.9	39.3 ± 4.5	0.683
Total Cholesterol (mg/dL)	176.8 ± 40.4	178.0 ± 38.4	175.9 ± 41.8	0.589	175.8 ± 42.0	177.3 ± 39.6	0.668
CRP (mg/dL)	4.90 ± 9.25	4.73 ± 9.83	5.06 ± 8.91	0.467	5.60 ± 10.55	4.49 ± 8.43	0.214
Death at 3 months (mRS = 6)	61 (26.7%)	30 (31.6%)	31 (23.5%)	0.175	23 (26.7%)	38 (26.8%)	0.998

Data are numbers (%) for categorical variables and mean ± SD for continuous variables except for NIHSS, which is median (IQR). *p*-values are derived from the chi-squared tests and the Mann-Whitney tests, and statistically significant values (*p* < 0.05) are indicated with bold and an asterisk (*). SBP: systolic blood pressure; mRS: modified Rankin Scale; NIHSS: National Institute of Health stroke scale; DBP: diastolic blood pressure; HR: heart rate; ABPM: ambulatory blood pressure monitoring; eGFR: estimated glomerular filtration rate; WBC: white blood cells count; CRP: C-reactive protein.

**Table 2:** Severity of stroke (NIHSS) and outcome at 3 months according to SBP circadian pattern.

All Stroke Patients						
SBP Circadian Pattern		Midday and Nocturnal Dipping	Midday Dipping and Nocturnal Rise	Midday Rise and Nocturnal Dipping	Midday and Nocturnal Rise	*p*
n = 228		n = 70	n = 62	n = 72	n = 23	
NIHSS admission	7 (13)	4 (10)	6 (12)	9 (14)	12 (11)	0.003 *
Death at 3 months	61 (26.7%)	14 (20.0%)	17 (27.4%)	24 (33.3%)	6 (26.1%)	0.358
Without prior disability						
n = 175		n = 58	n = 46	n = 53	n = 18	*p*
NIHSS admission	6 (9)	4 (8)	6 (12)	7 (9)	12 (11)	0.001 *
Disability/death	79 (45.1%)	17 (29.3%)	23 (50.0%)	25 (47.2%)	14 (77.8%)	0.003 *

Data are numbers (%) and NIHSS median (IQR), with *p*-values for the trend between the different SBP circadian pattern groups, derived from the chi-squared tests and the non-parametric Kruskal-Wallis tests; statistically significant values (*p* < 0.05) are indicated with bold and with an asterisk (*), NIHSS: National Institute of Health stroke scale; SBP: systolic blood pressure.

**Table 3:** Univariate logistic regression analysis of dipping status and SBP circadian patterns concerning the 3-month outcome.

Variables	Death Outcome		Unfavorable Outcome	
	Hazard Ratio (95% CI)	*p*	Hazard Ratio (95% CI)	*p*
Nocturnal SBP Dippers	1.00 (0.54–1.83)	0.998	0.44 (0.23–0.83)	0.011 *
Midday SBP Dippers	0.66 (0.36–1.20)	0.176	0.51 (0.27–0.94)	0.032 *
Nocturnal DBP Dippers	0.53 (0.29–0.97)	0.040 *	0.39 (0.21–0.75)	0.005 *
Midday DBP Dippers	0.75 (0.42–1.37)	0.360	0.59 (0.32–1.09)	0.095
Midday and Nocturnal Dippers (SBP)	0.59 (0.30–1.16)	0.128	0.36 (0.18–0.72)	0.004 *
Midday Dippers and Nocturnal Risers (SBP)	1.04 (0.54–2.01)	0.890	1.30 (0.66–2.56)	0.441
Midday Risers and Nocturnal Dippers (SBP)	1.60 (0.87–2.97)	0.129	1.12 (0.58–2.14)	0.723
Midday and Nocturnal Risers (SBP)	0.96 (0.36–2.56)	0.939	4.95 (1.56–15.73)	0.007 *

Univariate logistic regression analysis (ENTER method). *p*-values derived from the univariate analysis are reported, and statistically significant hazard ratios (*p* < 0.05) are indicated with bold and with an asterisk (*); CI: confidence interval; SBP: systolic blood pressure; DBP: diastolic blood pressure.

## Data Availability

The data that support the findings of this study are available from the corresponding author upon reasonable request.

## Supplementary Materials

The following supporting information can be downloaded at https://www.mdpi.com/article/10.3390/jcm12144816/s1. Table S1. Baseline clinical and laboratory findings and characteristics of study participants according to SBP dipping status. Table S2. Blood pressure and heart rate parameters derived from ABPM of study participants according to SBP dipping status. Table S3. Baseline clinical and laboratory findings and characteristics of study participants according to DBP dipping status. Table S4. Blood pressure and heart rate parameters derived from ABPM of study participants according to DBP dipping status. Table S5. Correlation analyses of midday BP dipping with nocturnal BP dipping in study participants. Table S6. Comparisons blood pressure and heart rate parameters derived from ABPM of study participants according to stroke severity (NIHSS). Table S7. Correlation analyses of NIHSS with midday and night SBP, midday SBP dipping and heart rate in study participants. Table S8. Baseline clinical and laboratory findings and characteristics of study participants according to SBP Circadian Pattern. Table S9. Comparisons blood pressure and heart rate parameters derived from ABPM of study participants according to SBP Circadian Pattern. Table S10. Baseline clinical and laboratory findings and characteristics of study participants without prior disability, according to SBP dipping status. Table S11. Blood pressure and heart rate parameters derived from ABPM of study participants without prior disability, according to SBP dipping status. Table S12. Baseline clinical and laboratory findings and characteristics of study participants without prior disability, according to DBP dipping status. Table S13. Blood pressure and heart rate parameters derived from ABPM of study participants without prior disability, according to DBP dipping status. Table S14. Correlation analyses of midday BP dipping with nocturnal BP dipping in study participants without prior disability. Table S15. Comparisons blood pressure and heart rate parameters derived from ABPM of study participants without prior disability, according to stroke severity (NIHSS). Table S16. Correlation analyses of NIHSS with midday SBP dipping and heart rate in study participants without prior disability. Table S17. Baseline clinical and laboratory findings and characteristics of study participants without prior disability, according to SBP Circadian Pattern. Table S18. Comparisons blood pressure and heart rate parameters derived from ABPM of study participants without prior disability, according to SBP Circadian Pattern. Figure S1. Relationships of midday BP dipping with nocturnal BP dipping of (A) systolic (SBP) and (B) diastolic (DBP) blood pressure derived from ABPM of all study participants. Figure S2. Circadian variation of mean systolic blood pressure (SBP) with 95% CI of all study participants in the four different SBP circadian pattern groups: (A) Midday and nocturnal dipping; (B) Midday dipping and nocturnal rise; (C) Midday rise and nocturnal dipping and (D) Midday and nocturnal rise. Figure S3. Relationships of midday BP dipping with nocturnal BP dipping of (A) systolic (SBP) and (B) diastolic (DBP) blood pressure derived from ABPM of study participants without prior disability. Figure S4. Circadian variation of mean systolic blood pressure (SBP) with 95% CI of study participants without prior disability in the four different SBP circadian pattern groups: (A) Midday and nocturnal dipping; (B) Midday dipping and nocturnal rise; (C) Midday rise and nocturnal dipping and (D) Midday and nocturnal rise.
