# Supplementary material for: Midday Dipping and Circadian Blood Pressure Patterns in Acute Ischemic Stroke
Source: J Clin Med. 2023 Jul 21;12(14):4816. doi: 10.3390/jcm12144816 (PMC10381256; doi:10.3390/jcm12144816)

Table S1. Baseline clinical and laboratory findings and characteristics of study participants according to SBP dipping status.

		Total (n=228)	Midday SBP dipping status			Nocturnal SBP dipping status		
			Non-dippers ≤0% (n=95)	Dippers >0% (n=132)	p	Non-dippers ≤0% (n=86)	Dippers >0% (n=142)	p
**Age (years)**		80.0±7.1	80.0±7.0	80.0±7.1	0.871	80.6±6.9	79.6±7.1	0.450
**Sex (male)**		104 (45.6%)	40 (42.1%)	64 (48.5%)	0.341	39 (45.3%)	65 (45.8%)	0.950
**BMI (Kg/m²)**		27.2±4.3	27.6±4.8	27.0±3.9	0.400	27.4±4.6	27.1±4.0	0.856
**Pre-Stroke mRS>2**		51 (22.6%)	24 (25.3%)	26 (20.0%)	0.348	20 (23.8%)	31 (21.8%)	0.731
**NIHSS admission**		7 (13)	10 (14)	5 (12)	**0.002***	9 (12)	6 (12)	0.179
**TOAST classification**	**LAA**	37 (16.2%)	14 (14.7%)	23 (17.4%)	0.242	15 (17.4%)	22 (15.5%)	0.961
	**CE**	81 (35.5%)	34 (35.8%)	47 (35.6%)		30 (34.9%)	51 (35.9%)	
	**LAC**	18 (7.9%)	4 (4.2%)	14 (10.6%)		6 (7.0%)	12 (8.5%)	
	**IUC**	92 (40.4%)	43 (45.3%)	48 (36.4%)		35 (40.7%)	57 (40.1%)	
**Hypertension**		191 (83.8%)	85 (89.5%)	105 (79.5%)	**0.046***	69 (80.2%)	122 (85.9%)	0.259
**Diabetes**		71 (31.1%)	28 (29.5%)	43 (32.6%)	0.619	24 (27.9%)	47 (33.1%)	0.412
**Dyslipidemia**		93 (40.8%)	44 (46.3%)	49 (37.1%)	0.165	31 (36.0%)	62 (43.7%)	0.257
**Atrial Fibrillation**		89 (39.0%)	39 (41.1%)	50 (37.9%)	0.629	38 (44.2%)	51 (35.9%)	0.215
**Coronary Artery Disease**		45 (19.7%)	17 (17.9%)	28 (21.2%)	0.536	20 (23.3%)	25 (17.6%)	0.299
**Heart Failure**		20 (8.8%)	8 (8.4%)	12 (9.1%)	0.861	5 (5.8%)	15 (10.6%)	0.219
**Previous Stroke**		86 (37.7%)	32 (33.7%)	54 (40.9%)	0.268	32 (37.2%)	54 (38.0%)	0.902
**Prosthetic Valves**		2 (0.9%)	2 (2.1%)	0 (0.0%)	0.174	0 (0.0%)	2 (1.4%)	0.387
**Cancer**		15 (6.6%)	5 (5.3%)	10 (7.6%)	0.489	4 (4.7%)	11 (7.7%)	0.361
**Smoking**		59 (25.9%)	21 (22.1%)	38 (28.8%)	0.257	24 (27.9%)	35 (24.6%)	0.586
**Thrombolysis**		7 (3.1%)	3 (3.2%)	4 (3.0%)	0.956	2 (2.3%)	5 (3.5%)	0.612
**Number of BP-lowering agents (pre-Stroke)**	**0**	37 (17.9%)	12 (14.5%)	25 (20.3%)	0.268	19 (24.4%)	18 (14.0%)	0.060
	**1**	58 (28.0%)	20 (24.1%)	37 (30.1%)		24 (30.8%)	34 (26.4%)	
	**2**	60 (29.0%)	30 (36.1%)	30 (24.4%)		15 (19.2%)	45 (34.9%)	
	**≥3**	52 (25.1%)	21 (25.3%)	31 (25.2%)		20 (25.6%)	32 (24.8%)	
**SBP admission (mmHg)**		154.6±27.0	162.0±29.3	149.8±23.9	**0.003***	155.7±26.7	154.0±27.2	0.887
**DBP admission (mmHg)**		84.3±16.2	87.3±17.3	82.2±15.2	**0.034***	86.1±18.0	83.2±15.1	0.363
**HR admission (bpm)**		77.7±15.6	79.5±16.7	76.4±14.6	0.307	78.1±15.5	77.4±15.6	0.714
**Onset-admission (hours)**		4.2±4.9	4.0±4.6	4.4±5.2	0.616	3.6±4.1	4.7±5.4	0.258
**Admission-ABPM(hours)**		14.1±8.7	12.9±8.5	14.9±8.7	0.084	14.6±8.9	13.9±8.5	0.466
**Onset-ABPM (hours)**		18.8±9.8	17.2±9.8	20.0±9.7	**0.037***	18.6±10.0	19.0±9.6	0.855
**Glucose (mg/dl)**		135.0±47.8	140.9±52.5	131.0±43.9	**0.046***	134.8±50.6	135.2±46.3	0.757
**Urea (mg/dl)**		49.6±27.2	48.5±26.9	50.5±27.6	0.344	45.8±20.0	51.6±30.3	0.370
**Creatinine (mg/dl)**		1.06±0.46	1.10±0.58	1.03±0.34	0.613	1.02±0.48	1.08±0.44	0.175
**eGFR (mL/min/1.73m²)**		63.1±19.4	61.3±19.0	64.3±19.6	0.290	65.2±18.6	61.9±19.8	0.204
**WBC (K/μl)**		8.6±3.0	9.3±3.4	8.2±2.5	**0.014***	8.2±2.1	8.9±3.4	0.621
**Hematocrit (%)**		39.4±4.6	39.3±4.3	39.5±4.9	0.663	39.7±4.9	39.3±4.5	0.683
**Hemoglobulin (g/dl)**		12.9±1.6	13.0±1.5	13.0±1.6	0.993	13.0±1.7	12.9±1.5	0.945
**Platelets (K/μl)**		234.8±102.2	234.5±122.2	235.3±85.9	0.364	235.2±102.4	234.6±102.5	0.785
**Total Cholesterol (mg/dl)**		176.8±40.4	178.0±38.4	175.9±41.8	0.589	175.8±42.0	177.3±39.6	0.668
**Triglycerides (mg/dl)**		117.8±62.6	117.7±70.0	117.9±57.0	0.743	115.2±63.0	119.3±62.6	0.416
**HDL (mg/dl)**		46.2±12.2	46.8±11.2	45.8±13.0	0.430	45.6±12.3	46.5±12.3	0.844
**LDL (mg/dl)**		108.8±37.2	108.5±32.3	109.0±40.6	0.629	108.0±43.3	109.2±33.3	0.418
**CRP (mg/dl)**		4.90±9.25	4.73±9.83	5.06±8.91	0.467	5.60±10.55	4.49±8.43	0.214
**Post-Stroke AHTs**		111 (49.1%)	46 (48.9%)	65 (49.6%)	0.920	38 (45.2%)	73 (51.4%)	0.370
**Death at 3 months(mRS=6)**		61 (26.7%)	30 (31.6%)	31 (23.5%)	0.175	23 (26.7%)	38 (26.8%)	0.998

Data are numbers (%) for categorical variables, mean ± SD for continuous variables except NIHSS which is median (IQR), p-values derived from the chi-squared tests and the Mann-Whitney tests and statistically significant values ($p < 0.05$) have been indicated bold with an asterisk (*), SBP: systolic blood pressure, BMI: body mass index, NIHSS: National Institute of Health stroke scale, LAA: large artery atherosclerotic stroke, CE: cardioembolic stroke, LAC: small artery occlusion or lacunar stroke, IUC: infarct of undetermined/multiple cause, ABPM: ambulatory blood pressure monitoring, mRS: modified Rankin Scale, DBP: diastolic blood pressure, HR: heart rate, eGFR: estimated glomerular filtration rate, WBC: white blood cells count, CRP: C-reactive protein, AHTs: antihypertensives.

Table S2. Blood pressure and heart rate parameters derived from ABPM of study participants according to SBP dipping status.

	Total (n=228)	Midday SBP dipping status			Nocturnal SBP dipping status		
		Non-dippers ≤0% (n=95)	Dippers >0% (n=132)	p	Non-dippers ≤0% (n=86)	Dippers >0% (n=142)	p
**Mean SBP_{24-h} (mmHg)**	149.8±19.9	152.2±22.0	147.9±18.1	0.240	154.0±20.3	147.2±19.3	**0.010***
**24-h SD_{SBP} (mmHg)**	17.9±6.4	17.8±6.3	18.0±6.4	0.807	18.3±6.8	17.6±6.1	0.421
**Mean SBP_{day} (mmHg)**	150.9±19.6	154.3±21.5	148.3±17.9	0.061	151.0±19.9	150.8±19.6	0.872
**Mean SBP_{night} (mmHg)**	147.8±22.8	148.4±24.9	147.0±21.1	0.949	159.8±22.3	140.5±19.9	**<0.001***
**Nocturnal SBP dipping (%)**	2.0±8.1	3.9±7.3	0.7±8.4	**0.002***	-5.8±5.8	6.8±5.0	**<0.001***
**Mean $SBP_{day\_without\_midday}$ (mmHg)**	151.4±19.6	152.2±21.6	150.6±18.1	0.790	152.7±19.8	150.6±19.5	0.431
**Mean SBP_{midday} (mmHg)**	149.7±22.8	161.4±22.9	141.4±18.9	**<0.001***	146.5±23.1	151.7±22.5	0.079
**Midday SBP dipping (%)**	1.0±8.1	-6.1±5.4	6.1±5.5	**<0.001***	3.8±8.2	-0.7±7.9	**<0.001***
**Mean DBP_{24-h} (mmHg)**	80.2±10.4	80.2±10.4	80.2±10.4	0.168	83.0±10.8	78.6±9.8	**0.003***
**24-h SD_{DBP} (mmHg)**	13.4±4.6	13.8±4.7	13.1±4.5	0.203	13.5±4.7	13.3±4.6	0.846
**Mean DBP_{day} (mmHg)**	81.0±10.3	82.8±10.3	79.7±10.1	**0.049***	81.9±10.7	80.4±10.0	0.529
**Mean DBP_{night} (mmHg)**	78.8±12.2	78.7±11.5	78.9±12.7	0.987	85.2±12.4	74.9±10.2	**<0.001***
**Nocturnal DBP dipping (%)**	2.6±8.9	4.7±8.3	1.0±9.1	**0.001***	-4.1±8.7	6.7±6.2	**0.001***
**Mean $DBP_{day\_without\_midday}$ (mmHg)**	81.2±10.4	82.0±10.7	80.7±10.3	0.566	82.6±10.9	80.3±10.1	0.241
**Mean DBP_{midday} (mmHg)**	80.1±12.2	85.5±11.7	76.2±11.1	**<0.001***	79.0±11.8	80.7±12.5	0.342
**Midday DBP dipping (%)**	1.2±10.4	-4.6±10.8	5.4±7.8	**<0.001***	4.2±8.6	-0.6±11.0	**0.003***
**Mean HR_{24-h} (bpm)**	73.3±13.8	76.0±13.3	71.5±13.9	**0.008***	76.6±15.1	71.3±12.6	**0.007***
**Mean HR_{day} (bpm)**	73.8±13.5	76.4±13.2	72.1±13.5	**0.011***	76.7±14.9	72.0±12.3	**0.018***
**Mean HR_{night} (bpm)**	72.3±15.1	75.1±14.2	70.5±15.3	**0.008***	76.3±16.5	69.9±13.7	**0.003***

Data are mean±SD, p-values derived from the Mann-Whitney tests and statistically significant values ($p < 0.05$) have been indicated bold with an asterisk (*), SBP: systolic blood pressure, SD: standard deviation, DBP: diastolic blood pressure, HR: heart rate.

Table S3. Baseline clinical and laboratory findings and characteristics of study participants according to DBP dipping status.

		Total (n=228)	Midday DBP dipping status			Nocturnal DBP dipping status		
			Non-dippers ≤0% (n=93)	Dippers >0% (n=134)	p	Non-dippers ≤0% (n=80)	Dippers >0% (n=148)	p
**Age (years)**		80.0±7.1	80.0±7.4	80.0±6.8	0.929	80.2±8.1	79.9±6.5	0.463
**Sex (male)**		104 (45.6%)	42 (45.2%)	62 (46.3%)	0.869	38 (47.5%)	66 (44.6%)	0.674
**BMI (Kg/m^2)**		27.2±4.3	27.1±4.9	27.3±3.8	0.421	26.8±4.4	27.4±4.2	0.268
**Pre-Stroke mRS>2**		51 (22.6%)	23 (24.7%)	27 (20.5%)	0.447	19 (24.1%)	32 (21.8%)	0.696
**NIHSS admission**		7 (13)	9 (14)	6 (12)	**0.037***	10.5 (16)	6 (10)	**0.011***
**TOAST classification**	**LAA**	37 (16.2%)	14 (15.1%)	23 (17.2%)	0.167	15 (18.8%)	22 (14.9%)	0.332
	**CE**	81 (35.5%)	31 (33.3%)	50 (37.3%)		31 (38.8%)	50 (33.8%)	
	**LAC**	18 (7.9%)	4 (4.3%)	14 (10.4%)		8 (10.0%)	10 (6.8%)	
	**IUC**	92 (40.4%)	44 (47.3%)	47 (35.1%)		26 (32.5%)	66 (44.6%)	
**Hypertension**		191 (83.8%)	83 (89.2%)	107 (79.9%)	0.059	63 (78.8%)	128 (86.5%)	0.131
**Diabetes**		71 (31.1%)	30 (32.3%)	41 (30.6%)	0.791	29 (36.3%)	42 (28.4%)	0.221
**Dyslipidemia**		93 (40.8%)	36 (38.7%)	57 (42.5%)	0.564	32 (40.0%)	61 (41.2%)	0.858
**Atrial Fibrillation**		89 (39.0%)	37 (39.8%)	52 (38.8%)	0.882	36 (45.0%)	53 (35.8%)	0.175
**Coronary Artery Disease**		45 (19.7%)	17 (18.3%)	28 (20.9%)	0.627	20 (25.0%)	25 (16.9%)	0.142
**Heart Failure**		20 (8.8%)	4 (4.3%)	16 (11.9%)	**0.046***	6 (7.5%)	14 (9.5%)	0.618
**Previous Stroke**		86 (37.7%)	26 (28.0%)	60 (44.8%)	**0.010***	27 (33.8%)	59 (39.9%)	0.363
**Prosthetic Valves**		2 (0.9%)	0 (0.0%)	2 (1.5%)	0.347	0 (0.0%)	2 (1.4%)	0.296
**Cancer**		15 (6.6%)	7 (7.5%)	8 (6.0%)	0.642	5 (6.3%)	10 (6.8%)	0.883
**Smoking**		59 (25.9%)	27 (29.0%)	32 (23.9%)	0.384	26 (32.5%)	33 (22.3%)	0.093
**Thrombolysis**		7 (3.1%)	5 (5.4%)	2 (1.5%)	0.096	4 (5.0%)	3 (2.0%)	0.214
**Number of BP-lowering agents (pre-Stroke)**	**0**	37 (17.9%)	10 (12.0%)	27 (22.0%)	0.309	20 (27.4%)	17 (12.7%)	**<0.001***
	**1**	58 (28.0%)	23 (27.7%)	34 (27.6%)		26 (35.6%)	32 (23.9%)	
	**2**	60 (29.0%)	27 (32.5%)	33 (26.8%)		9 (12.3%)	51 (38.1%)	
	**≥3**	52 (25.1%)	23 (27.7%)	29 (23.6%)		18 (24.7%)	34 (25.4%)	
**SBP admission (mmHg)**		154.6±27.0	160.2±28.1	151.1±25.5	**0.027***	158.0±28.8	152.8±25.9	0.269
**DBP admission (mmHg)**		84.3±16.2	85.9±17.3	83.2±15.4	0.232	87.7±17.2	82.4±15.4	**0.021***
**HR admission (bpm)**		77.7±15.6	79.0±15.9	76.8±15.4	0.441	79.3±15.4	76.8±15.6	0.232
**Onset-admission (hours)**		4.2±4.9	3.9±4.8	4.5±5.1	0.129	4.0±4.9	4.4±5.0	0.450
**Admission-ABPM(hours)**		14.1±8.7	12.5±8.4	15.2±8.7	**0.022***	14.5±9.0	13.9±8.5	0.622
**Onset-ABPM (hours)**		18.8±9.8	16.6±9.2	20.4±9.9	**0.004***	19.0±10.1	18.7±9.6	0.797
**Glucose (mg/dl)**		135.0±47.8	146.4±56.1	127.4±39.6	**0.005***	138.1±51.1	133.4±46.0	0.462
**Urea (mg/dl)**		49.6±27.2	47.8±24.1	50.9±29.3	0.550	45.7±17.0	51.6±31.0	0.854
**Creatinine (mg/dl)**		1.06±0.46	1.11±0.60	1.03±0.33	0.729	1.08±0.60	1.05±0.36	0.487
**eGFR (mL/min/1.73m^2)**		63.1±19.4	62.0±19.3	63.8±19.5	0.564	64.0±18.6	62.6±19.8	0.649
**WBC (K/μl)**		8.6±3.0	9.1±3.3	8.3±2.7	0.073	8.6±2.5	8.7±3.2	0.535
**Hematocrit (%)**		39.4±4.6	39.5±5.3	39.4±4.1	0.705	39.9±5.1	39.2±4.3	0.133
**Hemoglobulin (g/dl)**		12.9±1.6	12.9±1.9	13.0±1.3	0.906	13.1±1.7	12.9±1.5	0.250
**Platelets (K/μl)**		234.8±102.2	249.6±133.5	225.0±73.0	0.507	238.3±73.9	233.0±114.6	**0.037***
**Total Cholesterol (mg/dl)**		176.8±40.4	178.2±37.6	175.8±42.3	0.497	179.5±40.5	175.4±40.4	0.476
**Triglycerides (mg/dl)**		117.8±62.6	113.7±46.5	120.4±71.0	0.609	120.4±59.3	116.4±64.5	0.393
**HDL (mg/dl)**		46.2±12.2	45.7±10.7	46.5±13.2	0.934	45.9±14.2	46.4±11.1	0.433
**LDL (mg/dl)**		108.8±37.2	108.9±30.6	108.7±41.1	0.528	111.1±42.2	107.5±34.2	0.630
**CRP (mg/dl)**		4.90±9.25	5.61±10.24	4.48±8.63	0.184	4.56±5.51	5.08±10.74	0.248
**Post-Stroke AHTs**		111 (49.1%)	37 (39.8%)	74 (56.1%)	**0.016***	36 (45.6%)	75 (51.0%)	0.434
**Death at 3 months (mRS=6)**		61 (26.7%)	28 (30.1%)	33 (24.6%)	0.360	28 (35.0%)	33 (22.3%)	**0.039***

Data are numbers (%) for categorical variables, mean ± SD for continuous variables except NIHSS which is median (IQR), p-values derived from the chi-squared tests and the Mann-Whitney tests and statistically significant values ($p < 0.05$) have been indicated bold with an asterisk (*), DBP: diastolic blood pressure, BMI: body mass index, NIHSS: National Institute of Health stroke scale, LAA: large artery atherosclerotic stroke, CE: cardioembolic stroke, LAC: small artery occlusion or lacunar stroke, IUC: infarct of undetermined/multiple cause, ABPM: ambulatory blood pressure monitoring, mRS: modified Rankin Scale, DBP: diastolic blood pressure, HR: heart rate, eGFR: estimated glomerular filtration rate, WBC: white blood cells count, CRP: C-reactive protein, AHTs: antihypertensives.

Table S4. Blood pressure and heart rate parameters derived from ABPM of study participants according to DBP dipping status.

	Total (n=228)	Midday DBP dipping status			Nocturnal DBP dipping status		
		Non-dippers ≤0% (n=93)	Dippers >0% (n=134)	p	Non-dippers ≤0% (n=80)	Dippers >0% (n=148)	p
**Mean $SBP_{24\text{-}h}$ (mmHg)**	149.8±19.9	150.6±19.1	149.1±20.4	0.465	155.5±20.8	146.7±18.8	**0.001***
**24-h SD_{SBP} (mmHg)**	17.9±6.4	18.0±7.0	17.9±5.9	0.828	18.1±6.9	17.8±6.1	0.940
**Mean SBP_{day} (mmHg)**	150.9±19.6	152.4±19.4	149.6±19.8	0.280	153.3±20.8	149.5±19.0	0.171
**Mean SBP_{night} (mmHg)**	147.8±22.8	147.1±21.4	147.9±23.7	0.914	159.8±22.7	141.3±20.1	**<0.001***
**Nocturnal SBP dipping (%)**	2.0±8.1	3.3±8.1	1.1±8.0	0.053	-4.3±7.2	5.4±6.4	**<0.001***
**Mean $SBP_{day\_without\_midday}$ (mmHg)**	151.4±19.6	151.3±19.7	151.3±19.6	0.974	154.7±20.6	149.6±18.9	0.059
**Mean SBP_{midday} (mmHg)**	149.7±22.8	157.0±21.2	144.7±22.6	**<0.001***	149.3±24.1	150.0±22.2	0.841
**Midday SBP dipping (%)**	1.0±8.1	-3.9±7.2	4.4±6.9	**<0.001***	3.4±8.1	-0.3±7.9	**0.001***
**Mean $DBP_{24\text{-}h}$ (mmHg)**	80.2±10.4	80.1±9.2	80.4±11.2	0.808	84.9±11.0	77.7±9.2	**<0.001***
**24-h SD_{DBP} (mmHg)**	13.4±4.6	13.4±4.8	13.4±4.4	0.866	13.9±4.9	13.1±4.4	0.213
**Mean DBP_{day} (mmHg)**	81.0±10.3	81.3±9.7	80.7±10.7	0.548	83.0±11.0	79.9±9.7	0.085
**Mean DBP_{night} (mmHg)**	78.8±12.2	77.6±9.9	79.6±13.5	0.575	88.4±11.7	73.6±8.8	**<0.001***
**Nocturnal DBP dipping (%)**	2.6±8.9	4.4±7.9	1.3±9.4	**0.017***	-6.6±6.6	7.6±5.4	**<0.001***
**Mean $DBP_{day\_without\_midday}$ (mmHg)**	81.2±10.4	80.0±9.7	82.1±10.9	0.194	83.7±11.4	79.8±9.6	**0.039***
**Mean DBP_{midday} (mmHg)**	80.1±12.2	85.8±11.2	76.1±11.4	**<0.001***	80.8±11.8	79.7±12.5	0.534
**Midday DBP dipping (%)**	1.2±10.4	-7.4±9.2	7.2±6.1	**<0.001***	3.1±9.4	0.1±10.8	0.086
**Mean $HR_{24\text{-}h}$ (bpm)**	73.3±13.8	74.4±11.7	72.7±15.0	0.099	78.1±14.5	70.7±12.7	**<0.001***
**Mean HR_{day} (bpm)**	73.8±13.5	75.0±11.8	73.1±14.6	0.073	77.9±14.3	71.6±12.6	**0.001***
**Mean HR_{night} (bpm)**	72.3±15.1	73.1±12.4	72.0±16.6	0.188	78.7±15.6	68.9±13.6	**<0.001***

Data are mean±SD, p-values derived from the Mann-Whitney tests and statistically significant values ($p < 0.05$) have been indicated bold with an asterisk (*), SBP: systolic blood pressure, SD: standard deviation, DBP: diastolic blood pressure, HR: heart rate.

Table S5. Correlation analyses of midday BP dipping with nocturnal BP dipping in study participants.

correlation analysis	Systolic Blood Pressure		Diastolic Blood Pressure	
	Spearman's rank correlation coefficient	p value	Spearman's rank correlation coefficient	p value
**Midday dipping – Nocturnal dipping**	- 0.261	**<0.001***	- 0.174	**0.009***

p-values derived from the Spearman's rank correlation analysis and statistically significant values ($p < 0.05$) have been indicated bold with an asterisk (*), BP: blood pressure.

Table S6. Comparisons blood pressure and heart rate parameters derived from ABPM of study participants according to stroke severity (NIHSS).

	**Total (n=228)**	**NIHSS <8 (n=120)**	**NIHSS 8-16 (n=56)**	**NIHSS >16 (n=52)**	**p**
**Mean $SBP_{24\text{-}h}$ (mmHg)**	149.8±19.9	147.7±18.2	154.8±16.5	149.2±25.7	0.052
**24-h SD_{SBP} (mmHg)**	17.9±6.4	17.7±5.4	18.5±6.3	17.8±8.3	0.347
**Mean SBP_{day} (mmHg)**	150.9±19.6	149.3±18.4	155.5±16.3	149.5±24.7	0.113
**Mean SBP_{night} (mmHg)**	147.8±22.8	144.7±20.1	153.6±19.8	148.6±29.9	**0.037***
**Nocturnal SBP dipping (%)**	2.0±8.1	2.9±7.2	1.1±7.7	0.7±10.1	0.390
**Mean $SBP_{day\_without\_midday}$ (mmHg)**	151.4±19.6	150.2±18.5	155.7±16.1	149.5±24.7	0.146
**Mean SBP_{midday} (mmHg)**	149.7±22.8	146.8±21.2	155.9±19.8	150.1±29.2	**0.036***
**Midday SBP dipping (%)**	1.0±8.1	2.2±6.9	-0.2±6.8	-0.5±11.3	**0.008***
**Mean $DBP_{24\text{-}h}$ (mmHg)**	80.2±10.4	78.8±9.6	82.3±9.8	81.3±12.4	0.096
**24-h SD_{DBP} (mmHg)**	13.4±4.6	13.3±4.3	14.3±4.5	12.6±5.3	0.142
**Mean DBP_{day} (mmHg)**	81.0±10.3	79.8±9.6	83.1±9.7	81.4±12.2	0.210
**Mean DBP_{night} (mmHg)**	78.8±12.2	77.0±11.1	80.5±11.9	81.1±14.2	0.059
**Nocturnal DBP dipping (%)**	2.6±8.9	3.4±8.5	2.9±9.5	-2.1±9.2	0.086
**Mean $DBP_{day\_without\_midday}$ (mmHg)**	81.2±10.4	80.1±9.9	83.2±9.6	81.5±12.2	0.236
**Mean DBP_{midday} (mmHg)**	80.1±12.2	78.4±10.8	82.5±12.2	81.3±14.9	0.094
**Midday DBP dipping (%)**	1.2±10.4	1.8±9.3	0.8±10.1	-0.0±13.0	0.182
**Mean $HR_{24\text{-}h}$ (bpm)**	73.3±13.8	68.7±10.4	75.1±15.6	82.0±14.3	**<0.001***
**Mean HR_{day} (bpm)**	73.8±13.5	69.5±10.3	75.4±15.4	82.1±14.0	**<0.001***
**Mean HR_{night} (bpm)**	72.3±15.1	67.3±11.5	74.4±17.1	81.7±15.3	**<0.001***

Data are mean ± SD, p-values derived from the non-parametric Kruskal-Wallis tests and statistically significant values (p <0.05) have been indicated bold with an asterisk (*), NIHSS: National Institute of Health stroke scale, SBP: systolic blood pressure, DBP: diastolic blood pressure, HR: heart rate.

Table S7. Correlation analyses of NIHSS with midday and night SBP, midday SBP dipping and heart rate in study participants.

**correlation analysis**	Spearman’s rank correlation coefficient	p value
**NIHSS - SBP_{night}**	0.109	0.102
**NIHSS - SBP_{midday}**	0.095	0.152
**NIHSS - Midday SBP dipping**	- 0.193	**0.004***
**NIHSS - $HR_{24\text{-}h}$**	0.369	**<0.001***
**NIHSS - HR_{day}**	0.353	**<0.001***
**NIHSS - HR_{night}**	0.373	**<0.001***

p-values derived from the Spearman’s rank correlation analysis and statistically significant values (p <0.05) have been indicated bold with an asterisk (*), NIHSS: National Institute of Health stroke scale, SBP: systolic blood pressure, HR: heart rate.

Table S8. Baseline clinical and laboratory findings and characteristics of study participants according to SBP Circadian Pattern.

		Total (n=228)	SBP Circadian Pattern				p
			Midday&Nocturnal Dipping (n=70)	Midday Dipping & Nocturnal Rise (n=62)	Midday Rise & Nocturnal Dipping (n=72)	Midday&Nocturnal Rise (n=23)	
**Age (years)**		80.0±7.1	79.5±7.5	80.6±6.7	79.8±6.7	80.7±7.8	0.872
**Sex (male)**		104 (45.6%)	33 (47.1%)	31 (50.0%)	32 (44.4%)	8 (34.8%)	0.644
**BMI (Kg/m^2)**		27.2±4.3	26.7±3.2	27.3±4.6	27.5±4.7	27.7±5.0	0.795
**Pre-Stroke mRS>2**		51 (22.6%)	12 (17.1%)	14 (23.3%)	19 (26.4%)	5 (21.7%)	0.612
**NIHSS admission**		7 (13)	4 (10)	6 (12)	9 (14)	12 (11)	**0.003***
**TOAST classification**	**LAA**	37 (16.2%)	12 (17.1%)	11 (17.7%)	10 (13.9%)	4 (17.4%)	0.757
	**CE**	81 (35.5%)	24 (34.3%)	23 (37.1%)	27 (37.5%)	7 (30.4%)	
	**LAC**	18 (7.9%)	8 (11.4%)	6 (9.7%)	4 (5.6%)	0 (0.0%)	
	**IUC**	92 (40.4%)	26 (37.1%)	22 (35.5%)	31 (43.1%)	12 (52.2%)	
**Hypertension**		191(83.8%)	59 (84.3%)	46 (74.2%)	63 (87.5%)	22 (95.7%)	0.063
**Diabetes**		71 (31.1%)	24 (34.3%)	19 (30.6%)	23 (31.9%)	5 (21.7%)	0.730
**Dyslipidemia**		93 (40.8%)	27 (38.6%)	22 (35.5%)	35 (48.6%)	9 (39.1%)	0.439
**Atrial Fibrillation**		89 (39.0%)	23 (32.9%)	27 (43.5%)	28 (38.9%)	11 (47.8%)	0.495
**Coronary Artery Disease**		45 (19.7%)	13 (18.6%)	15 (24.2%)	12 (16.7%)	5 (21.7%)	0.725
**Heart Failure**		20 (8.8%)	8 (11.4%)	4 (6.5%)	7 (9.7%)	1 (4.3%)	0.643
**Previous Stroke**		86 (37.7%)	31 (44.3%)	23 (37.1%)	23 (31.9%)	9 (39.1%)	0.507
**Prosthetic Valves**		2 (0.9%)	0 (0.0%)	0 (0.0%)	2 (2.8%)	0 (0.0%)	0.227
**Cancer**		15 (6.6%)	7 (10.0%)	3 (4.8%)	4 (5.6%)	1 (4.3%)	0.585
**Smoking**		59 (25.9%)	19 (27.1%)	19 (30.6%)	16 (22.2%)	5 (21.7%)	0.684
**Thrombolysis**		7 (3.1%)	3 (4.3%)	1 (1.6%)	2 (2.8%)	1 (4.3%)	0.818
**Number of BP-lowering agents (pre-Stroke)**	**0**	37 (17.9%)	9 (14.1%)	16 (27.1%)	9 (13.8%)	3 (16.7%)	0.367
	**1**	58 (28.0%)	19 (29.7%)	18 (30.5%)	15 (23.1%)	5 (27.8%)	
	**2**	60 (29.0%)	20 (31.3%)	10 (16.9%)	25 (38.5%)	5 (27.8%)	
	**≥3**	52 (25.1%)	16 (25.0%)	15 (25.4%)	16 (24.6%)	5 (27.8%)	
**SBP admission (mmHg)**		154.6±27.0	146.4±25.1	153.6±21.9	161.4±27.3	163.8±35.9	**0.011***
**DBP admission (mmHg)**		84.3±16.2	79.6±15.2	85.1±14.8	86.6±14.2	89.5±25.4	**0.036***
**HR admission (bpm)**		77.7±15.6	74.7±13.6	78.4±15.6	80.0±17.0	77.5±16.1	0.411
**Onset-admission (hours)**		4.2±4.9	4.8±5.7	4.0±4.5	4.5±5.1	2.6±2.6	0.590
**Admission-ABPM(hours)**		14.1±8.7	15.0±8.8	14.9±8.7	12.8±8.2	13.4±9.5	0.382
**Onset-ABPM (hours)**		18.8±9.8	20.4±9.4	19.4±10.0	17.5±9.7	16.4±10.1	0.179
**Glucose (mg/dl)**		135.0±47.8	132.9±45.2	128.8±42.7	137.5±47.5	152.0±66.3	0.200
**Urea (mg/dl)**		49.6±27.2	53.5±31.9	46.8±21.0	49.9±28.9	43.6±18.2	0.521
**Creatinine (mg/dl)**		1.06±0.46	1.06±0.38	0.99±0.28	1.10±0.50	1.09±0.84	0.452
**eGFR (mL/min/1.73m^2)**		63.1±19.4	63.3±21.0	65.5±18.1	60.5±18.7	64.3±20.2	0.473
**WBC (K/μl)**		8.6±3.0	8.2±2.7	8.1±2.3	9.5±3.8	8.5±1.5	0.105
**Hematocrit (%)**		39.4±4.6	38.8±5.0	40.4±4.6	39.8±3.8	37.9±5.3	0.071
**Hemoglobulin (g/dl)**		12.9±1.6	12.7±1.7	13.3±1.4	13.1±1.3	12.5±2.2	0.387
**Platelets (K/μl)**		234.8±102.2	239.9±93.6	230.1±76.5	229.4±110.7	252.0±157.4	0.785
**Total Cholesterol (mg/dl)**		176.8±40.4	173.8±38.5	178.5±46.0	181.0±40.7	168.7±29.4	0.527
**Triglycerides (mg/dl)**		117.8±62.6	116.0±45.2	120.1±69.4	122.5±76.6	102.7±41.9	0.766
**HDL (mg/dl)**		46.2±12.2	47.3±13.1	44.0±12.8	45.8±11.5	49.9±10.1	0.321
**LDL (mg/dl)**		108.8±37.2	106.4±33.6	112.1±47.5	112.1±33.1	97.3±27.4	0.343
**CRP (mg/dl)**		4.90±9.25	4.92±10.78	5.22±6.18	4.08±5.35	6.86±18.10	0.350
**Post-Stroke AHTs**		111 (49.1%)	39 (55.7%)	26 (42.6%)	34 (47.2%)	12 (54.5%)	0.456
**Death at 3 months (mRS=6)**		61 (26.7%)	14 (20.0%)	17 (27.4%)	24 (33.3%)	6 (26.1%)	0.358

Data are numbers (%) for categorical variables, mean ± SD for continuous variables except NIHSS which is median (IQR), p-values for the trend between the different SBP Circadian Pattern groups, derived from the chi-squared tests and the non-parametric Kruskal-Wallis tests and statistically significant values (p <0.05) have been indicated bold with an asterisk (*), BMI: body mass index, NIHSS: National Institute of Health stroke scale, LAA: large artery atherosclerotic stroke, CE: cardioembolic stroke, LAC: small artery occlusion or lacunar stroke, IUC: infarct of undetermined/multiple cause, ABPM: ambulatory blood pressure monitoring, mRS: modified Rankin Scale, SBP: systolic blood pressure, DBP: diastolic blood pressure, HR: heart rate, ABPM: ambulatory blood pressure monitoring, eGFR: estimated glomerular filtration rate, WBC: white blood cells count, CRP: C-reactive protein, AHTs: antihypertensives.

Table S9. Comparisons blood pressure and heart rate parameters derived from ABPM of study participants according to SBP Circadian Pattern.

		**SBP Circadian Pattern**				
	**Total (n=228)**	**Midday&Nocturnal Dipping (n=70)**	**Midday Dipping & Nocturnal Rise (n=62)**	**Midday Rise & Nocturnal Dipping (n=72)**	**Midday&Nocturnal Rise (n=23)**	**p**
**Mean $SBP_{24\text{-}h}$ (mmHg)**	149.8±19.9	144.7±17.9	151.4±17.8	149.7±20.4	160.0±25.4	**0.024***
**24-h SD_{SBP} (mmHg)**	17.9±6.4	17.6±6.6	18.5±6.2	17.7±5.5	18.2±8.5	0.617
**Mean SBP_{day} (mmHg)**	150.9±19.6	148.1±18.1	148.5±17.7	153.4±20.7	157.1±24.3	0.266
**Mean SBP_{night} (mmHg)**	147.8±22.8	138.1±18.4	157.2±19.3	142.8±21.2	165.8±28.2	**<0.001***
**Nocturnal SBP dipping (%)**	2.0±8.1	6.7±4.7	-5.9±6.3	6.8±5.3	-5.3±4.6	**<0.001***
**Mean $SBP_{day\_without\_midday}$ (mmHg)**	151.4±19.6	150.0±18.4	151.3±17.9	151.2±20.7	155.6±24.4	0.836
**Mean SBP_{midday} (mmHg)**	149.7±22.8	142.7±19.7	139.9±17.9	160.4±21.9	164.4±26.1	**<0.001***
**Midday SBP dipping (%)**	1.0±8.1	4.9±4.4	7.4±6.3	-6.2±5.9	-5.7±3.6	**<0.001***
**Mean $DBP_{24\text{-}h}$ (mmHg)**	80.2±10.4	76.8±9.6	82.4±10.8	80.2±9.7	85.0±11.1	**0.003***
**24-h SD_{DBP} (mmHg)**	13.4±4.6	12.7±4.6	13.6±4.4	13.9±4.5	13.5±5.3	0.334
**Mean DBP_{day} (mmHg)**	81.0±10.3	78.6±10.0	80.9±10.3	82.1±9.8	84.7±11.8	0.162
**Mean DBP_{night} (mmHg)**	78.8±12.2	73.2±9.8	85.2±12.7	76.6±10.5	85.3±12.0	**<0.001***
**Nocturnal DBP dipping (%)**	2.6±8.9	6.7±5.8	-5.3±7.8	6.7±6.6	-1.3±10.3	**<0.001***
**Mean $DBP_{day\_without\_midday}$ (mmHg)**	81.2±10.4	79.4±9.9	82.1±10.5	81.2±10.2	84.4±12.0	0.520
**Mean DBP_{midday} (mmHg)**	80.1±12.2	75.9±11.5	76.6±10.7	85.4±11.6	85.6±12.3	**<0.001***
**Midday DBP dipping (%)**	1.2±10.4	4.5±7.4	6.4±8.2	-5.6±11.6	-1.6±6.8	**<0.001***
**Mean $HR_{24\text{-}h}$ (bpm)**	73.3±13.8	67.6±11.2	76.0±15.3	74.9±12.8	79.2±14.4	**<0.001***
**Mean HR_{day} (bpm)**	73.8±13.5	68.7±11.2	75.9±14.9	75.3±12.6	79.8±14.8	**0.001***
**Mean HR_{night} (bpm)**	72.3±15.1	65.4±11.9	76.2±16.8	74.3±13.9	77.9±15.2	**<0.001***

Data are mean±SD, p-values for the trend between the different SBP Circadian Pattern groups, derived from the non-parametric Kruskal-Wallis tests and statistically significant values ($p < 0.05$) have been indicated bold with an asterisk (*), SBP: systolic blood pressure, SD: standard deviation, DBP: diastolic blood pressure, HR: heart rate.

Figure S1. Relationships of midday BP dipping with nocturnal BP dipping of (A) systolic (SBP) and (B) diastolic (DBP) blood pressure derived from ABPM of all study participants.

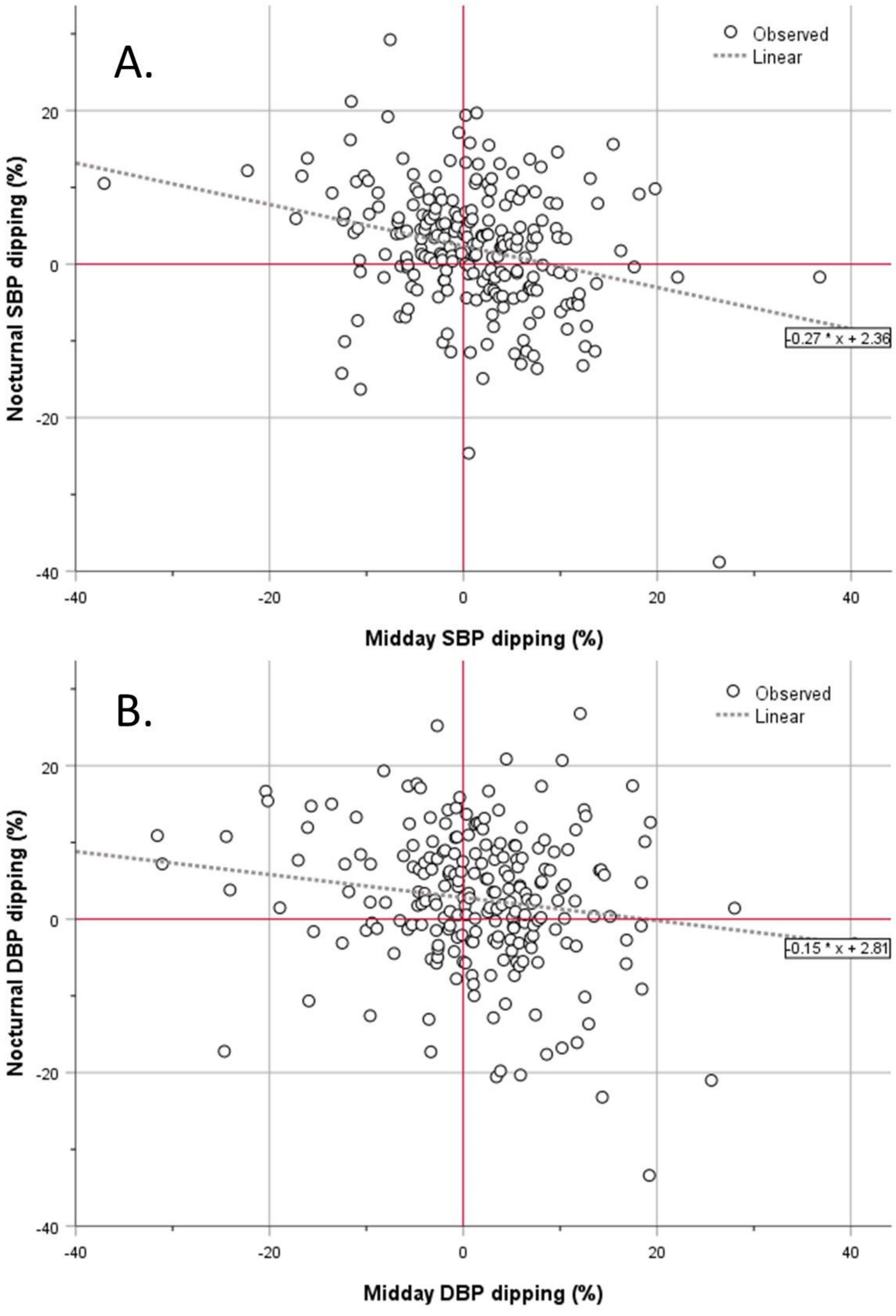

Figure S2. Circadian variation of mean systolic blood pressure (SBP) with 95% CI of all study participants in the four different SBP circadian pattern groups: (A) Midday and nocturnal dipping; (B) Midday dipping and nocturnal rise; (C) Midday rise and nocturnal dipping and (D) Midday and nocturnal rise. Yellow: Midday time (13:00-16:59); Red: Night time (23:00-6:59); Red dashed line: Mean systolic blood pressure of all study participants without prior disability (149.8 mmHg).

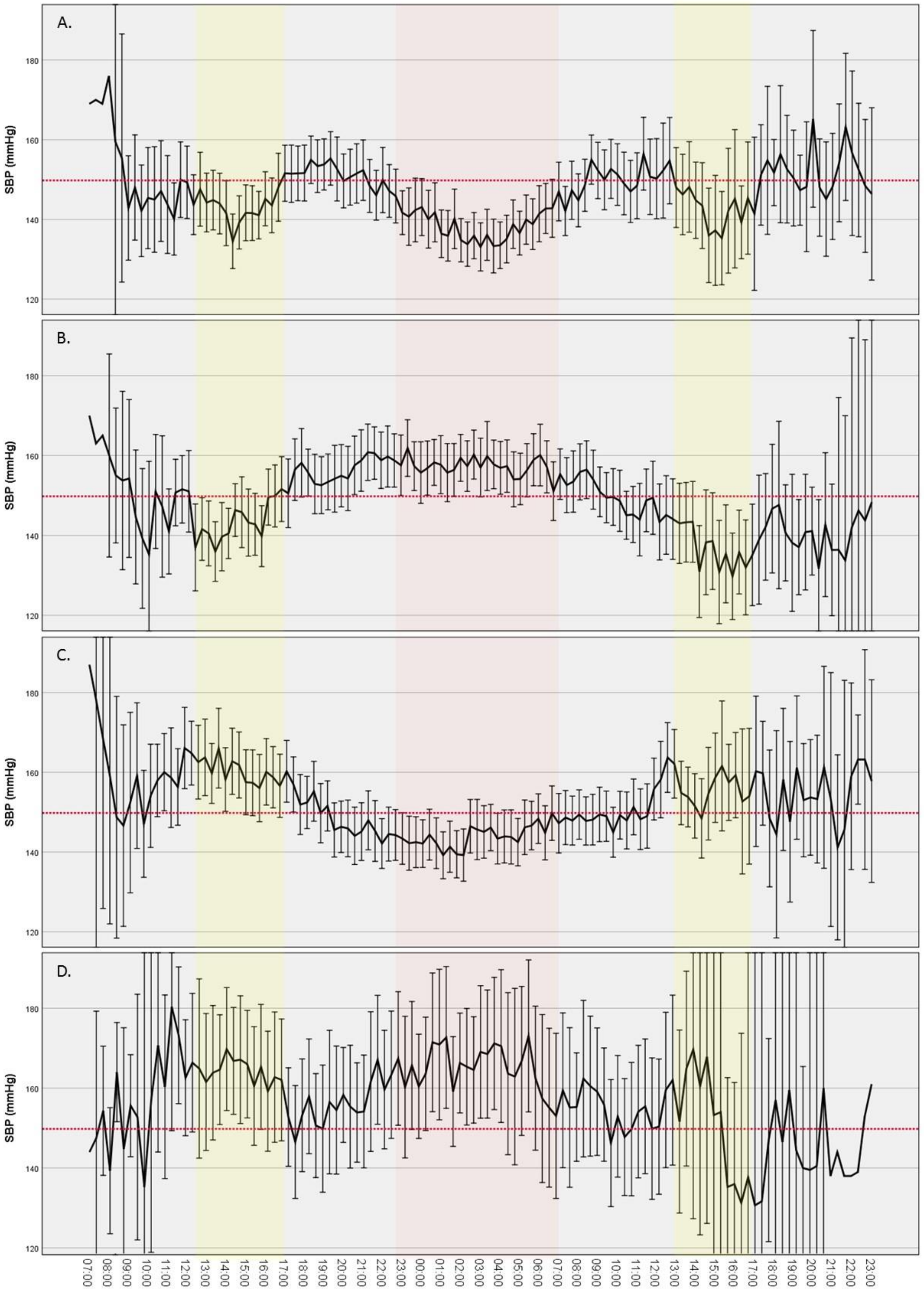

Supplement: Supplementary file 1 [file jcm-12-04816-s001.zip › Supplementary S1.pdf]
