# Supplementary material for: Midday Dipping and Circadian Blood Pressure Patterns in Acute Ischemic Stroke
Source: J Clin Med. 2023 Jul 21;12(14):4816. doi: 10.3390/jcm12144816 (PMC10381256; doi:10.3390/jcm12144816)

Table S10. Baseline clinical and laboratory findings and characteristics of study participants without prior disability, according to SBP dipping status.

		Total (n=175)	Midday SBP dipping status			Nocturnal SBP dipping status		
			Non-dippers ≤0% (n=71)	Dippers >0% (n=104)	p	Non-dippers ≤0% (n=64)	Dippers >0% (n=111)	p
**Age (years)**		78.7±6.9	79.2±6.9	78.4±7.0	0.481	79.1±6.6	78.5±7.1	0.669
**Sex (male)**		89 (50.9%)	36 (50.7%)	53 (51.0%)	0.973	31 (48.4%)	58 (52.3%)	0.627
**BMI (Kg/m²)**		27.1±3.9	27.2±3.8	27.1±4.0	0.934	27.5±5.1	27.0±3.1	0.866
**NIHSS admission**		6 (9)	9 (9)	4 (8)	**<0.001***	7 (12)	5 (9)	0.126
**TOAST classification**	**LAA**	31 (17.7%)	10 (14.1%)	21 (20.2%)	0.115	13 (20.3%)	18 (16.2%)	0.865
	**CE**	58 (33.1%)	23 (32.4%)	35 (33.7%)		22 (34.4%)	36 (32.4%)	
	**LAC**	18 (10.3%)	4 (5.6%)	14 (13.5%)		6 (9.4%)	12 (10.8%)	
	**IUC**	68 (38.9%)	34 (47.9%)	34 (32.7%)		23 (35.9%)	45 (40.5%)	
**Hypertension**		151 (86.3%)	65 (91.5%)	86 (82.7%)	0.094	53 (82.8%)	98 (88.3%)	0.310
**Diabetes**		57 (32.6%)	20 (28.2%)	37 (35.6%)	0.305	21 (32.8%)	36 (32.4%)	0.959
**Dyslipidemia**		78 (44.6%)	36 (50.7%)	42 (40.4%)	0.177	24 (37.5%)	54 (48.6%)	0.153
**Atrial Fibrillation**		64 (36.6%)	27 (38.0%)	37 (35.6%)	0.741	27 (42.2%)	37 (33.3%)	0.241
**Coronary Artery Disease**		36 (20.6%)	13 (18.3%)	23 (22.1%)	0.541	15 (23.4%)	21 (18.9%)	0.476
**Heart Failure**		14 (8.0%)	4 (5.6%)	10 (9.6%)	0.340	4 (6.3%)	10 (9.0%)	0.517
**Previous Stroke**		56 (32.0%)	18 (25.4%)	38 (36.5%)	0.119	21 (32.8%)	35 (31.5%)	0.861
**Prosthetic Valves**		1 (0.6%)	1 (1.4%)	0 (0.0%)	0.406	0 (0.0%)	1 (0.9%)	0.634
**Cancer**		6 (3.4%)	2 (2.8%)	4 (3.8%)	0.713	2 (3.1%)	4 (3.6%)	0.867
**Smoking**		52 (29.7%)	19 (26.8%)	33 (31.7%)	0.480	20 (31.3%)	32 (28.8%)	0.736
**Thrombolysis**		7 (4.0%)	3 (4.2%)	4 (3.8%)	0.900	2 (3.1%)	5 (4.5%)	0.654
**Number of BP-lowering agents (pre-Stroke)**	**0**	30 (18.6%)	9 (14.1%)	21 (21.6%)	0.486	14 (24.6%)	16 (15.4%)	0.089
	**1**	42 (26.1%)	17 (26.6%)	25 (25.8%)		16 (28.1%)	26 (25.0%)	
	**2**	44 (27.3%)	21 (32.8%)	23 (23.7%)		9 (15.8%)	35 (33.7%)	
	**≥3**	45 (28.0%)	17 (26.6%)	28 (28.9%)		18 (31.6%)	27 (26.0%)	
**SBP admission (mmHg)**		154.9±25.4	160.5±28.2	151.1±22.8	**0.038***	157.8±24.3	153.2±26.0	0.263
**DBP admission (mmHg)**		84.1±15.2	86.2±15.3	82.8±15.0	0.097	85.4±16.9	83.4±14.1	0.353
**HR admission (bpm)**		77.3±14.8	78.5±16.4	76.4±13.7	0.755	77.6±15.2	77.0±14.6	0.817
**Onset-admission (hours)**		4.3±5.0	4.3±5.0	4.3±5.1	0.909	3.2±3.8	4.8±5.6	0.131
**Admission-ABPM(hours)**		13.8±8.9	12.5±8.8	14.7±8.9	0.102	14.3±9.2	13.5±8.8	0.502
**Onset-ABPM (hours)**		18.5±9.9	17.0±10.2	19.5±9.6	0.097	18.0±10.0	18.8±9.9	0.619
**Glucose (mg/dl)**		136.3±46.9	140.3±50.3	133.6±44.5	0.220	137.2±52.1	135.9±44.1	0.969
**Urea (mg/dl)**		49.0±24.5	49.4±23.7	48.7±24.1	0.868	47.5±21.2	49.8±26.3	0.815
**Creatinine (mg/dl)**		1.07±0.48	1.15±0.65	1.02±0.32	0.272	1.05±0.54	1.09±0.45	0.380
**eGFR (mL/min/1.73m²)**		63.8±19.3	61.3±19.5	65.6±19.0	0.186	65.2±19.6	63.1±19.2	0.473
**WBC (K/μl)**		8.4±2.8	9.1±3.3	8.0±2.4	**0.019***	8.1±1.9	8.6±3.2	0.919
**Hematocrit (%)**		39.8±4.5	39.5±4.4	40.0±4.5	0.456	39.5±5.2	39.9±4.0	0.485
**Hemoglobulin (g/dl)**		13.1±1.5	13.0±1.6	13.2±1.4	0.586	12.9±1.8	13.2±1.3	0.263
**Platelets (K/μl)**		233.8±80.7	223.6±96.3	223.9±68.5	0.471	233.9±107.5	218.3±61.2	0.496
**Total Cholesterol (mg/dl)**		178.4±39.0	177.4±37.0	179.0±40.5	0.965	178.8±41.6	178.1±37.7	0.964
**Triglycerides (mg/dl)**		121.9±60.8	121.3±65.8	122.3±57.7	0.993	123.9±65.0	120.8±58.6	0.749
**HDL (mg/dl)**		45.7±11.6	45.8±10.1	45.7±12.6	0.774	45.1±12.4	46.1±11.2	0.593
**LDL (mg/dl)**		108.6±34.0	107.8±31.7	109.1±35.6	0.995	107.3±34.9	109.3±33.7	0.679
**CRP (mg/dl)**		4.89±10.13	4.76±10.75	4.99±9.72	0.891	6.15±12.03	4.20±8.92	0.138
**Post-Stroke AHTs**		89 (51.4%)	34 (48.6%)	55 (53.4%)	0.533	30 (48.4%)	59 (53.2%)	0.548
**Disability/death at 3 months (mRS>2)**		79 (45.1%)	39 (54.9%)	40 (38.5%)	**0.032***	37 (57.8%)	42 (37.8%)	**0.011***

Data are numbers (%) for categorical variables, mean ± SD for continuous variables except NIHSS which is median (IQR), p-values derived from the chi-squared tests and the Mann-Whitney tests and statistically significant values (p <0.05) have been indicated bold with an asterisk (*), SBP: systolic blood pressure, BMI: body mass index, NIHSS: National Institute of Health stroke scale, LAA: large artery atherosclerotic stroke, CE: cardioembolic stroke, LAC: small artery occlusion or lacunar stroke, IUC: infarct of undetermined/multiple cause, ABPM: ambulatory blood pressure monitoring, mRS: modified Rankin Scale, DBP: diastolic blood pressure, HR: heart rate, eGFR: estimated glomerular filtration rate, WBC: white blood cells count, CRP: C-reactive protein, AHTs: antihypertensives.

Table S11. Blood pressure and heart rate parameters derived from ABPM of study participants without prior disability, according to SBP dipping status.

	Total (n=175)	Midday SBP dipping status			Nocturnal SBP dipping status		
		Non-dippers ≤0% (n=71)	Dippers >0% (n=104)	p	Non-dippers ≤0% (n=64)	Dippers >0% (n=111)	p
**Mean $SBP_{24\text{-}h}$ (mmHg)**	150.5±19.1	151.6±21.5	149.8±17.3	0.751	153.1±20.8	149.1±18.0	0.121
**24-h SD_{SBP} (mmHg)**	17.7±6.1	17.7±5.7	17.8±6.3	0.624	17.5±6.1	17.9±6.1	0.743
**Mean SBP_{day} (mmHg)**	151.8±18.9	153.6±21.1	150.6±17.2	0.428	150.3±20.0	152.7±18.3	0.494
**Mean SBP_{night} (mmHg)**	148.1±22.0	147.9±24.6	148.2±20.1	0.635	158.6±23.1	142.0±18.8	**<0.001***
**Nocturnal SBP dipping (%)**	2.4±7.8	3.7±7.8	1.5±7.7	**0.033***	-5.4±4.7	7.0±5.1	**<0.001***
**Mean $SBP_{day\_without\_midday}$ (mmHg)**	152.4±18.9	151.7±21.1	152.9±17.4	0.566	151.7±20.0	152.9±18.4	0.744
**Mean SBP_{midday} (mmHg)**	150.9±21.5	160.4±22.6	144.4±18.2	**<0.001***	147.3±22.6	153.0±20.7	0.107
**Midday SBP dipping (%)**	0.9±7.1	-5.7±4.5	5.5±4.5	**<0.001***	2.9±7.1	-0.1±6.9	**0.005***
**Mean $DBP_{24\text{-}h}$ (mmHg)**	80.4±10.0	80.2±9.6	80.5±10.3	0.880	82.8±11.3	79.0±8.9	0.031*
**24-h SD_{DBP} (mmHg)**	13.4±4.2	13.7±4.3	13.3±4.1	0.247	13.1±4.2	13.6±4.2	0.254
**Mean DBP_{day} (mmHg)**	81.3±9.8	81.6±10.0	81.1±9.7	0.866	81.7±10.9	81.1±9.2	0.884
**Mean DBP_{night} (mmHg)**	78.5±11.9	77.3±10.8	79.4±12.6	0.337	84.6±13.2	75.1±9.6	**<0.001***
**Nocturnal DBP dipping (%)**	3.3±8.7	4.9±9.2	2.2±8.3	**0.029***	-3.4±8.1	7.3±6.4	**<0.001***
**Mean $DBP_{day\_without\_midday}$ (mmHg)**	81.6±10.0	80.9±10.2	82.1±9.8	0.342	82.4±11.2	81.1±9.2	0.708
**Mean DBP_{midday} (mmHg)**	80.3±11.4	84.0±11.4	77.7±10.8	**0.001***	79.3±11.2	80.8±11.6	0.529
**Midday DBP dipping (%)**	1.4±9.7	-4.0±10.2	5.2±7.1	**<0.001***	3.5±7.7	0.2±10.5	0.084
**Mean $HR_{24\text{-}h}$ (bpm)**	71.3±12.6	73.9±12.4	69.6±12.5	**0.024***	74.9±14.5	69.3±10.9	**0.011***
**Mean HR_{day} (bpm)**	71.9±12.4	74.3±12.6	70.3±12.1	**0.041***	75.2±14.3	70.0±10.8	**0.018***
**Mean HR_{night} (bpm)**	70.1±13.8	72.9±13.2	68.2±13.9	**0.014***	74.3±15.8	67.8±11.9	**0.008***

Data are mean±SD, p-values derived from the Mann-Whitney tests and statistically significant values (p <0.05) have been indicated bold with an asterisk (*), SBP: systolic blood pressure, SD: standard deviation, DBP: diastolic blood pressure, HR: heart rate.

Table S12. Baseline clinical and laboratory findings and characteristics of study participants without prior disability, according to DBP dipping status.

		Total (n=175)	Midday DBP dipping status			Nocturnal DBP dipping status		
			Non-dippers ≤0% (n=70)	Dippers >0% (n=105)	p	Non-dippers ≤0% (n=60)	Dippers >0% (n=115)	p
**Age (years)**		78.7±6.9	79.0±7.5	78.6±6.6	0.576	78.4±8.2	78.9±6.2	0.886
**Sex (male)**		89 (50.9%)	36 (51.4%)	53 (50.5%)	0.902	29 (48.3%)	60 (52.2%)	0.630
**BMI (Kg/m²)**		27.1±3.9	26.7±3.8	27.4±4.0	0.235	26.8±4.4	27.3±3.6	0.347
**NIHSS admission**		6 (9)	7 (9)	5 (10)	0.106	8.5 (14)	5 (8)	**0.030***
**TOAST classification**	**LAA**	31 (17.7%)	11 (15.7%)	20 (19.0%)	0.121	13 (21.7%)	18 (15.7%)	0.312
	**CE**	58 (33.1%)	21 (30.0%)	37 (35.2%)		21 (35.0%)	37 (32.2%)	
	**LAC**	18 (10.3%)	4 (5.7%)	14 (13.3%)		8 (13.3%)	10 (8.7%)	
	**IUC**	68 (38.9%)	34 (48.6%)	34 (32.4%)		18 (30.0%)	50 (43.5%)	
**Hypertension**		151 (86.3%)	63 (90.0%)	88 (83.8%)	0.244	47 (78.3%)	104 (90.4%)	**0.027***
**Diabetes**		57 (32.6%)	23 (32.9%)	34 (32.4%)	0.947	25 (41.7%)	32 (27.8%)	0.064
**Dyslipidemia**		78 (44.6%)	27 (38.6%)	51 (48.6%)	0.192	26 (43.3%)	52 (45.2%)	0.812
**Atrial Fibrillation**		64 (36.6%)	26 (37.1%)	38 (36.2%)	0.898	24 (40.0%)	40 (34.8%)	0.496
**Coronary Artery Disease**		36 (20.6%)	13 (18.6%)	23 (21.9%)	0.593	14 (23.3%)	22 (19.1%)	0.514
**Heart Failure**		14 (8.0%)	2 (2.9%)	12 (11.4%)	**0.041***	3 (5.0%)	11 (9.6%)	0.291
**Previous Stroke**		56 (32.0%)	13 (18.6%)	43 (41.0%)	**0.002***	16 (26.7%)	40 (34.8%)	0.275
**Prosthetic Valves**		1 (0.6%)	0 (0.0%)	1 (1.0%)	0.600	0 (0.0%)	1 (0.9%)	0.657
**Cancer**		6 (3.4%)	3 (4.3%)	3 (2.9%)	0.611	2 (3.3%)	4 (3.5%)	0.960
**Smoking**		52 (29.7%)	24 (34.3%)	28 (26.7%)	0.280	22 (36.7%)	30 (26.1%)	0.146
**Thrombolysis**		7 (4.0%)	5 (7.1%)	2 (1.9%)	0.083	4 (6.7%)	3 (2.6%)	0.193
**Number of BP-lowering agents (pre-Stroke)**	**0**	30 (18.6%)	9 (14.1%)	21 (21.6%)	0.603	17 (30.9%)	13 (12.3%)	**<0.001***
	**1**	42 (26.1%)	19 (29.7%)	23 (23.7%)		18 (32.7%)	24 (22.6%)	
	**2**	44 (27.3%)	17 (26.6%)	27 (27.8%)		5 (9.1%)	39 (36.8%)	
	**≥3**	45 (28.0%)	19 (29.7%)	26 (26.8%)		15 (27.3%)	30 (28.3%)	
**SBP admission (mmHg)**		154.9±25.4	158.4±27.7	152.5±23.6	0.223	157.2±28.2	153.6±23.9	0.400
**DBP admission (mmHg)**		84.1±15.2	84.8±15.4	83.6±15.1	0.377	86.4±15.9	82.9±14.7	0.112
**HR admission (bpm)**		77.3±14.8	79.1±15.4	76.0±14.4	0.277	78.5±14.4	76.6±15.1	0.457
**Onset-admission (hours)**		4.3±5.0	4.0±5.1	4.4±5.0	0.238	3.7±4.8	4.6±5.1	0.164
**Admission-ABPM(hours)**		13.8±8.9	11.5±8.5	15.4±8.9	**0.003***	14.1±9.2	13.7±8.8	0.815
**Onset-ABPM (hours)**		18.5±9.9	15.5±9.2	20.5±9.9	**0.002***	18.2±9.9	18.7±9.9	0.770
**Glucose (mg/dl)**		136.3±46.9	147.9±55.5	128.7±38.6	**0.024***	141.4±55.2	133.7±42.1	0.508
**Urea (mg/dl)**		49.0±24.5	49.1±26.1	48.9±23.6	0.567	46.6±17.2	50.3±27.7	0.820
**Creatinine (mg/dl)**		1.07±0.48	1.15±0.67	1.02±0.30	0.551	1.09±0.68	1.06±0.34	0.204
**eGFR (mL/min/1.73m²)**		63.8±19.3	62.3±20.2	64.9±18.7	0.531	65.7±20.1	62.9±18.9	0.281
**WBC (K/μl)**		8.4±2.8	8.9±3.3	8.1±2.4	0.092	8.3±2.3	8.5±3.0	0.703
**Hematocrit (%)**		39.8±4.5	40.1±5.1	39.6±4.0	0.591	39.7±5.0	39.8±4.2	0.648
**Hemoglobulin (g/dl)**		13.1±1.5	13.2±1.8	13.1±1.2	0.805	13.0±1.7	13.1±1.4	0.872
**Platelets (K/μl)**		233.8±80.7	227.7±99.4	221.1±66.1	0.963	230.6±61.5	220.4±88.8	**0.046***
**Total Cholesterol (mg/dl)**		178.4±39.0	179.4±37.5	177.8±40.1	0.641	178.1±40.0	178.6±38.7	0.919
**Triglycerides (mg/dl)**		121.9±60.8	120.6±48.4	122.7±67.2	0.390	126.1±61.7	119.7±60.5	0.491
**HDL (mg/dl)**		45.7±11.6	45.2±9.8	46.1±12.6	1.000	44.6±13.5	46.4±10.5	0.172
**LDL (mg/dl)**		108.6±34.0	109.6±31.0	108.0±35.9	0.648	107.2±33.7	109.3±34.3	0.847
**CRP (mg/dl)**		4.89±10.13	5.61±11.14	4.43±9.46	0.214	4.23±5.77	5.24±11.82	0.494
**Post-Stroke AHTs**		89 (51.4%)	26 (37.1%)	63 (61.2%)	**0.002***	28 (47.5%)	61 (53.5%)	0.450
**Disability/death at 3 months (mRS>2)**		79 (45.1%)	37 (52.9%)	42 (40.0%)	0.094	36 (60.0%)	43 (37.4%)	**0.004***

Data are numbers (%) for categorical variables, mean ± SD for continuous variables except NIHSS which is median (IQR), p-values derived from the chi-squared tests and the Mann-Whitney tests and statistically significant values (p <0.05) have been indicated bold with an asterisk (*), DBP: diastolic blood pressure, BMI: body mass index, NIHSS: National Institute of Health stroke scale, LAA: large artery atherosclerotic stroke, CE: cardioembolic stroke, LAC: small artery occlusion or lacunar stroke, IUC: infarct of undetermined/multiple cause, ABPM: ambulatory blood pressure monitoring, mRS: modified Rankin Scale.Data are mean±SD, p-values derived from the Mann-Whitney tests and statistically significant values (p <0.05) have been indicated bold with an asterisk (*), SBP: systolic blood pressure, DBP: diastolic blood pressure, HR: heart rate, ABPM: ambulatory blood pressure monitoring, eGFR: estimated glomerular filtration rate, WBC: white blood cells count, CRP: C-reactive protein, AHTs: antihypertensives.

Table S13. Blood pressure and heart rate parameters derived from ABPM of study participants without prior disability, according to DBP dipping status.

	Total (n=175)	Midday DBP dipping status			Nocturnal DBP dipping status		
		Non-dippers ≤0% (n=70)	Dippers >0% (n=105)	p	Non-dippers ≤0% (n=60)	Dippers >0% (n=115)	p
**Mean $SBP_{24\text{-}h}$ (mmHg)**	150.5±19.1	150.6±17.9	150.5±20.0	0.784	156.3±20.8	147.5±17.6	**0.003***
**24-h SD_{SBP} (mmHg)**	17.7±6.1	17.8±6.6	17.6±5.7	0.953	17.6±6.7	17.8±5.8	0.486
**Mean SBP_{day} (mmHg)**	151.8±18.9	152.4±18.6	151.5±19.2	0.711	154.3±20.3	150.5±18.1	0.217
**Mean SBP_{night} (mmHg)**	148.1±22.0	147.2±20.0	148.7±23.2	0.959	160.1±23.1	141.8±18.5	**<0.001***
**Nocturnal SBP dipping (%)**	2.4±7.8	3.1±8.7	1.9±7.1	0.245	-3.7±6.3	5.6±6.4	**<0.001***
**Mean $SBP_{day\_without\_midday}$ (mmHg)**	152.4±18.9	151.5±18.8	153.0±19.1	0.665	155.5±20.3	150.8±18.1	0.119
**Mean SBP_{midday} (mmHg)**	150.9±21.5	156.6±20.2	147.2±21.6	**0.005***	151.8±23.2	150.5±20.7	0.671
**Midday SBP dipping (%)**	0.9±7.1	-3.4±6.5	3.9±5.9	**<0.001***	2.4±7.1	0.2±7.1	0.066
**Mean $DBP_{24\text{-}h}$ (mmHg)**	80.4±10.0	79.9±8.8	80.7±10.8	0.989	85.3±11.5	77.8±8.1	**<0.001***
**24-h SD_{DBP} (mmHg)**	13.4±4.2	13.4±4.2	13.4±4.1	0.690	13.6±4.3	13.3±4.1	0.855
**Mean DBP_{day} (mmHg)**	81.3±9.8	81.2±9.5	81.4±10.1	0.899	83.6±11.3	80.1±8.8	0.094
**Mean DBP_{night} (mmHg)**	78.5±11.9	77.2±9.3	79.4±13.4	0.754	88.6±12.4	73.3±7.6	**<0.001***
**Nocturnal DBP dipping (%)**	3.3±8.7	4.5±8.6	2.5±8.8	0.117	-6.0±5.6	8.2±5.5	**<0.001***
**Mean $DBP_{day\_without\_midday}$ (mmHg)**	81.6±10.0	79.9±9.4	82.7±10.2	0.115	84.2±11.7	80.3±8.7	0.083
**Mean DBP_{midday} (mmHg)**	80.3±11.4	85.4±10.7	76.8±10.7	**<0.001***	81.8±11.5	79.5±11.4	0.264
**Midday DBP dipping (%)**	1.4±9.7	-7.0±8.5	7.1±5.3	**<0.001***	2.5±8.4	0.9±10.3	0.253
**Mean $HR_{24\text{-}h}$ (bpm)**	71.3±12.6	72.6±11.7	70.5±13.1	0.152	76.0±13.5	68.9±11.4	**0.001***
**Mean HR_{day} (bpm)**	71.9±12.4	73.3±12.0	71.0±12.7	0.134	75.9±13.3	69.9±11.5	**0.003***
**Mean HR_{night} (bpm)**	70.1±13.8	71.2±12.2	69.5±14.7	0.206	76.1±14.7	67.0±12.2	**<0.001***

Data are mean±SD, p-values derived from the Mann-Whitney tests and statistically significant values (p <0.05) have been indicated bold with an asterisk (*), SBP: systolic blood pressure, SD: standard deviation, DBP: diastolic blood pressure, HR: heart rate.

Table S14. Correlation analyses of midday BP dipping with nocturnal BP dipping in study participants without prior disability.

correlation analysis	Systolic Blood Pressure		Diastolic Blood Pressure	
	Spearman's rank correlation coefficient	p value	Spearman's rank correlation coefficient	p value
**Midday dipping – Nocturnal dipping**	- 0.194	**0.010***	- 0.120	0.113

p-values derived from the Spearman's rank correlation analysis and statistically significant values (p <0.05) have been indicated bold with an asterisk (*), BP: blood pressure.

Table S15. Comparisons blood pressure and heart rate parameters derived from ABPM of study participants without prior disability, according to stroke severity (NIHSS).

	Total (n=175)	NIHSS <8 (n=102)	NIHSS 8-16 (n=42)	NIHSS >16 (n=31)	p
**Mean $SBP_{24\text{-}h}$ (mmHg)**	150.5±19.1	148.5±18.8	153.8±15.4	153.0±23.9	0.137
**24-h SD_{SBP} (mmHg)**	17.7±6.1	17.6±5.5	18.1±5.9	17.6±8.2	0.624
**Mean SBP_{day} (mmHg)**	151.8±18.9	150.3±19.1	154.5±15.3	153.4±22.5	0.316
**Mean SBP_{night} (mmHg)**	148.1±22.0	145.0±20.5	152.6±18.4	152.2±29.0	0.096
**Nocturnal SBP dipping (%)**	2.4±7.8	3.4±7.4	1.2±7.4	0.9±9.3	0.328
**Mean $SBP_{day\_without\_midday}$ (mmHg)**	152.4±18.9	151.2±19.2	154.8±15.0	153.4±22.8	0.472
**Mean SBP_{midday} (mmHg)**	150.9±21.5	147.7±21.0	155.0±18.4	156.2±25.4	0.056
**Midday SBP dipping (%)**	0.9±7.1	2.3±6.6	-0.1±6.5	-1.9±8.5	**0.005***
**Mean $DBP_{24\text{-}h}$ (mmHg)**	80.4±10.0	79.5±9.8	80.5±9.3	83.1±11.2	0.296
**24-h SD_{DBP} (mmHg)**	13.4±4.2	13.3±4.0	14.1±4.2	13.1±4.7	0.547
**Mean DBP_{day} (mmHg)**	81.3±9.8	80.6±9.7	81.4±9.2	83.4±10.9	0.462
**Mean DBP_{night} (mmHg)**	78.5±11.9	77.3±11.5	78.7±11.6	82.4±13.2	0.156
**Nocturnal DBP dipping (%)**	3.3±8.7	4.0±8.4	3.2±9.7	1.1±8.3	0.242
**Mean $DBP_{day\_without\_midday}$ (mmHg)**	81.6±10.0	81.0±10.0	81.6±9.0	83.6±11.0	0.497
**Mean DBP_{midday} (mmHg)**	80.3±11.4	78.9±10.6	80.8±11.6	83.9±13.4	0.205
**Midday DBP dipping (%)**	1.4±9.7	2.3±8.5	0.9±9.9	-0.6±12.4	0.259
**Mean $HR_{24\text{-}h}$ (bpm)**	71.3±12.6	68.1±10.5	71.4±12.0	81.9±14.0	**<0.001***
**Mean HR_{day} (bpm)**	71.9±12.4	69.0±10.5	71.7±12.1	82.1±13.8	**<0.001***
**Mean HR_{night} (bpm)**	70.1±13.8	66.3±11.4	70.9±13.5	81.8±15.0	**<0.001***

Data are mean ± SD, p-values derived from the non-parametric Kruskal-Wallis tests and statistically significant values ($p < 0.05$) have been indicated bold with an asterisk (*), NIHSS: National Institute of Health stroke scale, SBP: systolic blood pressure, DBP: diastolic blood pressure, HR: heart rate.

Table S16. Correlation analyses of NIHSS with midday SBP dipping and heart rate in study participants without prior disability.

**correlation analysis**	Spearman's rank correlation coefficient	p value
**NIHSS - Midday SBP dipping**	- 0.250	**<0.001***
**NIHSS - $HR_{24\text{-}h}$**	0.317	**<0.001***
**NIHSS - HR_{day}**	0.288	**<0.001***
**NIHSS - HR_{night}**	0.341	**<0.001***

p-values derived from the Spearman's rank correlation analysis and statistically significant values ($p < 0.05$) have been indicated bold with an asterisk (*), NIHSS: National Institute of Health stroke scale, SBP: systolic blood pressure, HR: heart rate.

Table S17. Baseline clinical and laboratory findings and characteristics of study participants without prior disability, according to SBP Circadian Pattern.

		Total (n=175)	SBP Circadian Pattern				p
			**Midday&Nocturnal Dipping (n=58)**	**Midday Dipping & Nocturnal Rise (n=46)**	**Midday Rise & Nocturnal Dipping (n=53)**	**Midday&Nocturnal Rise (n=18)**	
**Age (years)**		78.7±6.9	77.9±7.3	79.1±6.6	79.2±7.0	79.1±6.8	0.634
**Sex (male)**		89 (50.9%)	30 (51.7%)	23 (50.0%)	28 (52.8%)	8 (44.4%)	0.938
**BMI (Kg/m²)**		27.1±3.9	27.0±3.1	27.3±5.0	26.9±3.1	28.0±5.7	0.995
**NIHSS admission**		6 (9)	4 (8)	6 (12)	7 (9)	12 (11)	**0.001***
**TOAST classification**	**LAA**	31 (17.7%)	12 (20.7%)	9 (19.6%)	6 (11.3%)	4 (22.2%)	0.563
	**CE**	58 (33.1%)	19 (32.8%)	16 (34.8%)	17 (32.1%)	6 (33.3%)	
	**LAC**	18 (10.3%)	8 (13.8%)	6 (13.0%)	4 (7.5%)	0 (0.0%)	
	**IUC**	68 (38.9%)	19 (32.8%)	15 (32.6%)	26 (49.1%)	8 (44.4%)	
**Hypertension**		151(86.3%)	51 (87.9%)	35 (76.1%)	47 (88.7%)	18 (100%)	0.063
**Diabetes**		57 (32.6%)	21 (36.2%)	16 (34.8%)	15 (28.3%)	5 (27.8%)	0.782
**Dyslipidemia**		78 (44.6%)	25 (43.1%)	17 (37.0%)	29 (54.7%)	7 (38.9%)	0.311
**Atrial Fibrillation**		64 (36.6%)	18 (31.0%)	19 (41.3%)	19 (35.8%)	8 (44.4%)	0.636
**Coronary Artery Disease**		36 (20.6%)	12 (20.7%)	11 (23.9%)	9 (17.0%)	4 (22.2%)	0.858
**Heart Failure**		14 (8.0%)	7 (12.1%)	3 (6.5%)	3 (5.7%)	1 (5.6%)	0.576
**Previous Stroke**		56 (32.0%)	23 (39.7%)	15 (32.6%)	12 (22.6%)	6 (33.3%)	0.294
**Prosthetic Valves**		1 (0.6%)	0 (0.0%)	0 (0.0%)	1 (1.9%)	0 (0.0%)	0.510
**Cancer**		6 (3.4%)	3 (5.2%)	1 (2.2%)	1 (1.9%)	1 (5.6%)	0.711
**Smoking**		52 (29.7%)	18 (31.0%)	15 (32.6%)	14 (26.4%)	5 (27.8%)	0.910
**Thrombolysis**		7 (4.0%)	3 (5.2%)	1 (2.2%)	2 (3.8%)	1 (5.6%)	0.867
**Number of BP-lowering agents (pre-Stroke)**	**0**	30 (18.6%)	9 (16.7%)	12 (27.9%)	7 (14.0%)	2 (14.3%)	0.499
	**1**	42 (26.1%)	13 (24.1%)	12 (27.9%)	13 (26.0%)	4 (28.6%)	
	**2**	44 (27.3%)	17 (31.5%)	6 (14.0%)	18 (36.0%)	3 (21.4%)	
	**≥3**	45 (28.0%)	15 (27.8%)	13 (30.2%)	12 (24.0%)	5 (35.7%)	
**SBP admission (mmHg)**		154.9±25.4	147.8±23.6	155.3±21.2	159.2±27.4	164.7±31.1	0.072
**DBP admission (mmHg)**		84.1±15.2	80.7±13.9	85.3±16.1	86.3±13.9	85.6±19.7	0.135
**HR admission (bpm)**		77.3±14.8	75.4±13.1	77.6±14.4	78.8±16.1	77.7±17.7	0.915
**Onset-admission (hours)**		4.3±5.0	5.0±5.7	3.4±4.1	4.7±5.5	3.0±2.7	0.437
**Admission-ABPM(hours)**		13.8±8.9	14.6±9.1	14.9±8.8	12.4±8.4	12.8±10.2	0.421
**Onset-ABPM (hours)**		18.5±9.9	20.1±9.6	18.7±9.6	17.3±10.0	16.2±11.0	0.321
**Glucose (mg/dl)**		136.3±46.9	136.9±46.9	129.1±41.1	134.7±41.2	157.7±70.4	0.340
**Urea (mg/dl)**		49.0±24.5	49.5±25.9	47.7±22.0	50.1±27.0	47.0±19.3	0.991
**Creatinine (mg/dl)**		1.07±0.48	1.04±0.34	0.99±0.30	1.13±0.55	1.20±0.94	0.643
**eGFR (mL/min/1.73m²)**		63.8±19.3	65.0±19.0	66.2±19.2	61.1±19.2	62.2±21.1	0.570
**WBC (K/μl)**		8.4±2.8	8.0±2.6	7.9±2.0	9.2±3.6	8.7±1.5	0.121
**Hematocrit (%)**		39.8±4.5	39.8±4.4	40.2±4.8	40.1±3.7	37.4±5.8	0.088
**Hemoglobulin (g/dl)**		13.1±1.5	13.2±1.4	13.2±1.4	13.2±1.3	12.3±2.4	0.246
**Platelets (K/μl)**		233.8±80.7	224.4±71.2	223.4±65.6	211.7±47.9	262.9±178.7	0.754
**Total Cholesterol (mg/dl)**		178.4±39.0	177.5±36.8	181.0±45.3	178.9±39.2	172.9±29.7	0.916
**Triglycerides (mg/dl)**		121.9±60.8	119.0±43.6	126.5±71.9	122.8±72.3	116.4±40.7	0.983
**HDL (mg/dl)**		45.7±11.6	47.3±12.5	43.7±12.6	44.8±9.6	49.0±11.6	0.495
**LDL (mg/dl)**		108.6±34.0	108.2±34.8	110.3±37.0	110.5±32.6	99.2±27.7	0.718
**CRP (mg/dl)**		4.89±10.13	4.67±11.51	5.40±6.95	3.73±5.31	8.00±20.03	0.447
**Post-Stroke AHTs**		89 (51.4%)	35 (60.3%)	20 (44.4%)	24 (45.3%)	10 (58.8%)	0.273
**Disability/death at 3 months (mRS>2)**		79 (45.1%)	17 (29.3%)	23 (50.0%)	25 (47.2%)	14 (77.8%)	**0.003***

Data are numbers (%) for categorical variables, mean ± SD for continuous variables except NIHSS which is median (IQR), p-values for the trend between the different SBP Circadian Pattern groups, derived from the chi-squared tests and the non-parametric Kruskal-Wallis tests and statistically significant values (p <0.05) have been indicated bold with an asterisk (*), BMI: body mass index, NIHSS: National Institute of Health stroke scale, LAA: large artery atherosclerotic stroke, CE: cardioembolic stroke, LAC: small artery occlusion or lacunar stroke, IUC: infarct of undetermined/multiple cause, ABPM: ambulatory blood pressure monitoring, mRS: modified Rankin Scale, SBP: systolic blood pressure, DBP: diastolic blood pressure, HR: heart rate, eGFR: estimated glomerular filtration rate, WBC: white blood cells count, CRP: C-reactive protein, AHTs: antihypertensives.

Table S18. Comparisons blood pressure and heart rate parameters derived from ABPM of study participants without prior disability, according to SBP Circadian Pattern.

	Total (n=175)	SBP Circadian Pattern				p
		Midday&Nocturnal Dipping (n=58)	Midday Dipping & Nocturnal Rise (n=46)	Midday Rise & Nocturnal Dipping (n=53)	Midday&Nocturnal Rise (n=18)	
**Mean $SBP_{24\text{-}h}$ (mmHg)**	150.5±19.1	148.4±15.8	151.5±19.2	149.7±20.3	157.2±24.7	0.392
**24-h SD_{SBP} (mmHg)**	17.7±6.1	18.0±6.8	17.5±5.8	17.8±5.2	17.4±7.2	0.951
**Mean SBP_{day} (mmHg)**	151.8±18.9	152.0±15.9	148.9±18.8	153.5±20.6	154.0±23.0	0.792
**Mean SBP_{night} (mmHg)**	148.1±22.0	141.5±16.9	156.7±20.7	142.6±20.9	163.6±28.4	**<0.001***
**Nocturnal SBP dipping (%)**	2.4±7.8	6.9±4.8	-5.2±4.7	7.0±5.5	-5.9±5.0	**<0.001***
**Mean $SBP_{day\_without\_midday}$ (mmHg)**	152.4±18.9	154.1±16.2	151.5±19.0	151.6±20.7	152.2±22.9	0.895
**Mean SBP_{midday} (mmHg)**	150.9±21.5	146.6±17.7	141.7±18.6	160.0±21.6	161.5±26.0	**<0.001***
**Midday SBP dipping (%)**	0.9±7.1	4.8±4.2	6.4±4.7	-5.6±4.8	-6.0±3.6	**<0.001***
**Mean $DBP_{24\text{-}h}$ (mmHg)**	80.4±10.0	78.9±8.7	82.7±11.7	79.2±9.3	83.0±10.4	0.188
**24-h SD_{DBP} (mmHg)**	13.4±4.2	13.5±4.3	13.0±3.9	13.8±4.0	13.3±5.0	0.504
**Mean DBP_{day} (mmHg)**	81.3±9.8	80.9±8.8	81.4±10.9	81.2±9.6	82.6±11.3	0.993
**Mean DBP_{night} (mmHg)**	78.5±11.9	74.8±9.5	85.1±13.8	75.3±9.9	83.3±11.5	**<0.001***
**Nocturnal DBP dipping (%)**	3.3±8.7	7.4±5.7	-4.2±6.2	7.1±7.1	-1.4±11.6	**<0.001***
**Mean $DBP_{day\_without\_midday}$ (mmHg)**	81.6±10.0	81.8±8.7	82.5±11.2	80.5±9.8	82.3±11.6	0.816
**Mean DBP_{midday} (mmHg)**	80.3±11.4	77.8±11.0	77.6±10.8	84.1±11.5	83.6±11.4	**0.009***
**Midday DBP dipping (%)**	1.4±9.7	4.9±7.5	5.7±6.6	-4.8±11.0	-1.8±7.6	**<0.001***
**Mean $HR_{24\text{-}h}$ (bpm)**	71.3±12.6	66.9±11.0	73.0±13.5	71.9±10.3	79.8±16.1	**0.002***
**Mean HR_{day} (bpm)**	71.9±12.4	68.0±10.9	73.1±12.9	72.3±10.3	80.4±16.7	**0.007***
**Mean HR_{night} (bpm)**	70.1±13.8	64.6±11.6	72.8±15.2	71.2±11.4	78.1±16.8	**0.001***

Data are mean±SD, p-values for the trend between the different SBP Circadian Pattern groups, derived from the non-parametric Kruskal-Wallis tests and statistically significant values (p <0.05) have been indicated bold with an asterisk (*), SBP: systolic blood pressure, SD: standard deviation, DBP: diastolic blood pressure, HR: heart rate.

Figure S3. Relationships of midday BP dipping with nocturnal BP dipping of (A) systolic (SBP) and (B) diastolic (DBP) blood pressure derived from ABPM of study participants without prior disability.

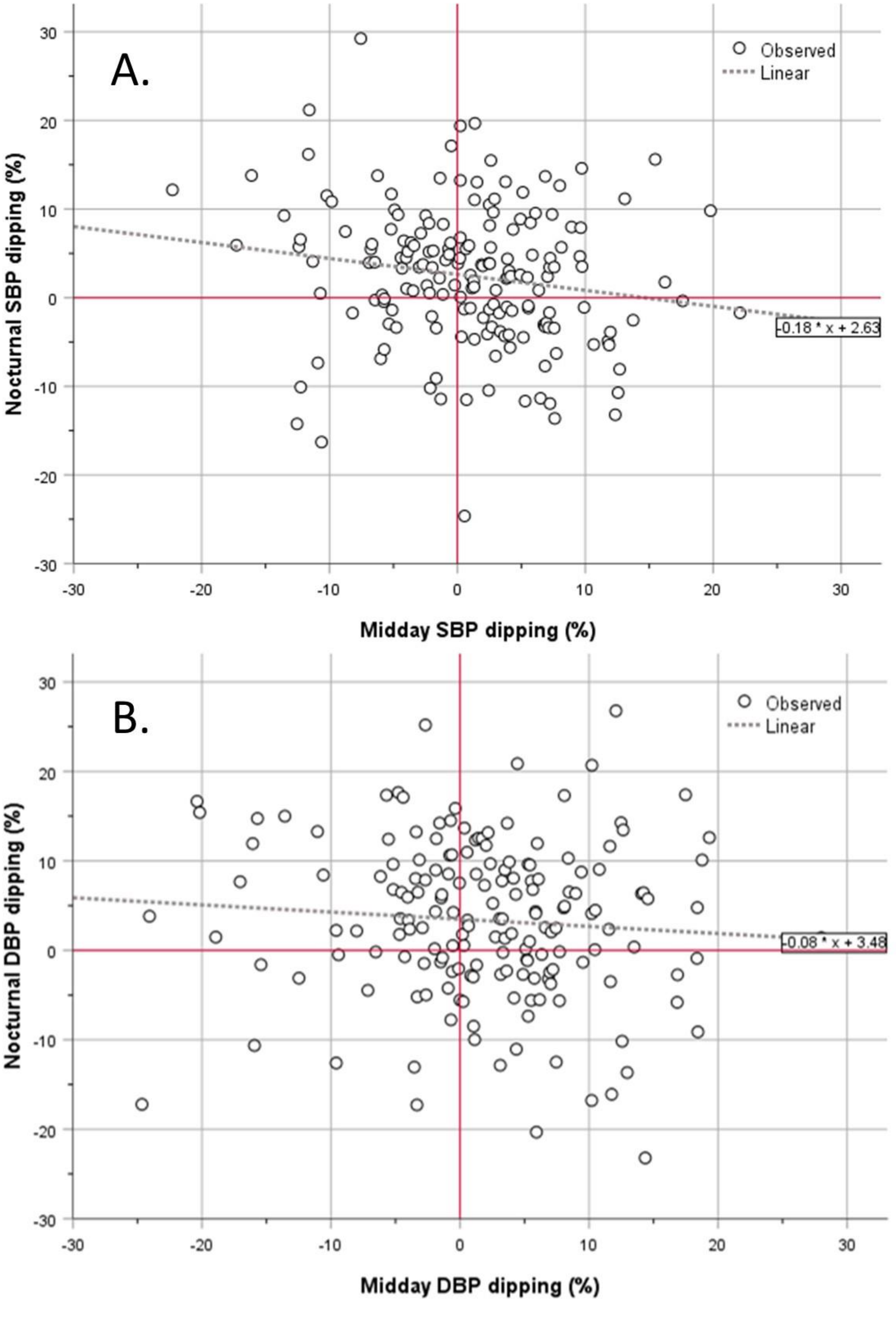

Figure S4. Circadian variation of mean systolic blood pressure (SBP) with 95% CI of study participants without prior disability in the four different SBP circadian pattern groups: (A) Midday and nocturnal dipping; (B) Midday dipping and nocturnal rise; (C) Midday rise and nocturnal dipping and (D) Midday and nocturnal rise. Yellow: Midday time (13:00-16:59); Red: Night time (23:00-6:59); Red dashed line: Mean systolic blood pressure of all study participants without prior disability (150.5 mmHg).

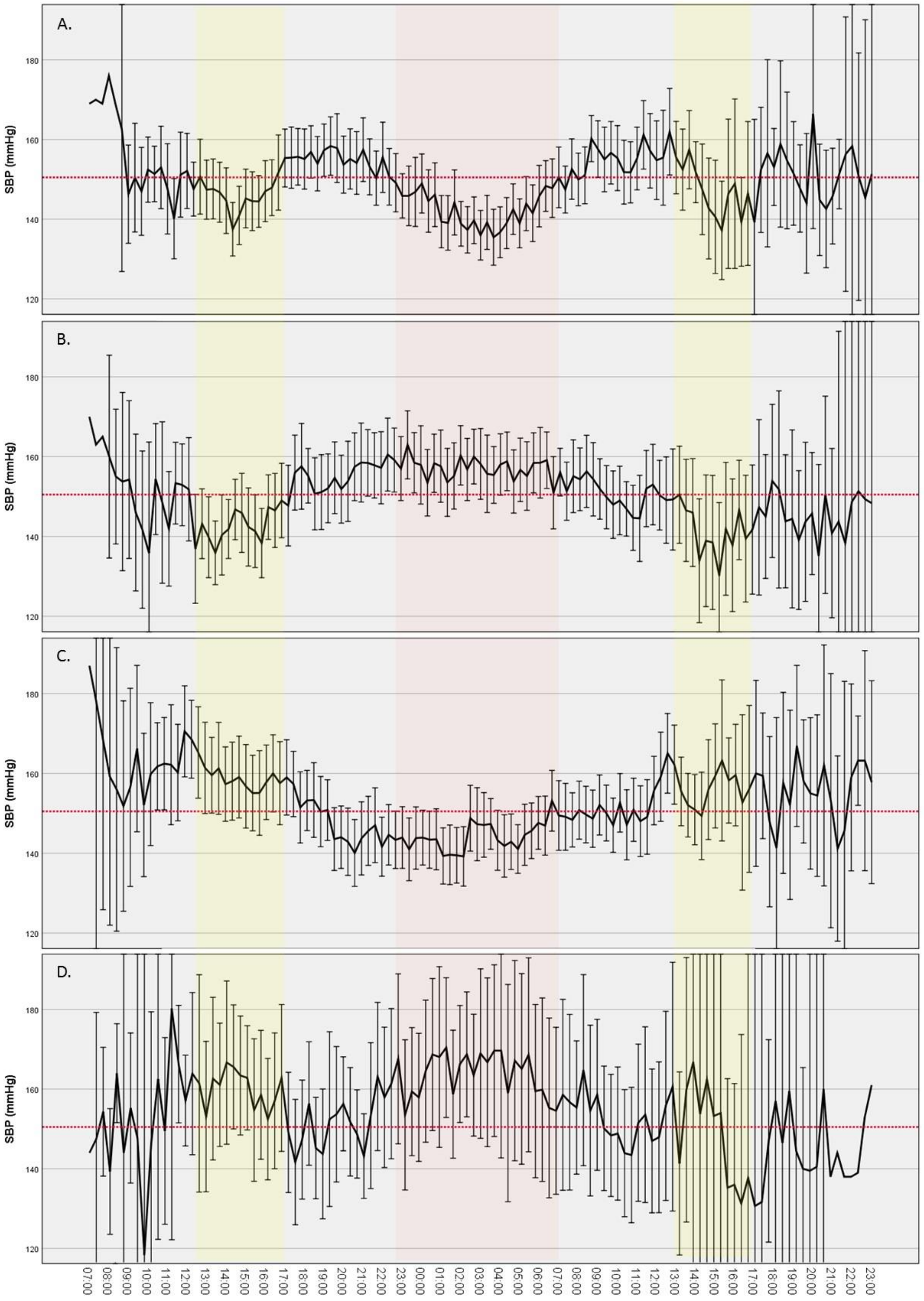

Supplement: Supplementary file 1 [file jcm-12-04816-s001.zip › Supplementary S2.pdf]
